# Does Amount of Information Support Aesthetic Values?

**DOI:** 10.3389/fnins.2022.805658

**Published:** 2022-03-22

**Authors:** Norberto M. Grzywacz, Hassan Aleem

**Affiliations:** ^1^Department of Psychology, Loyola University Chicago, Chicago, IL, United States; ^2^Department of Molecular Pharmacology and Neuroscience, Loyola University Chicago, Chicago, IL, United States; ^3^Interdisciplinary Program in Neuroscience, Georgetown University, Washington, DC, United States

**Keywords:** aesthetic value, value function, expected utility hypothesis, Shannon entropy, Fisher information, Kalman filtering (KF), surprise and expectation, survival-relevant information

## Abstract

Obtaining information from the world is important for survival. The brain, therefore, has special mechanisms to extract as much information as possible from sensory stimuli. Hence, given its importance, the amount of available information may underlie aesthetic values. Such information-based aesthetic values would be significant because they would compete with others to drive decision-making. In this article, we ask, “What is the evidence that amount of information support aesthetic values?” An important concept in the measurement of informational volume is entropy. Research on aesthetic values has thus used Shannon entropy to evaluate the contribution of quantity of information. We review here the concepts of information and aesthetic values, and research on the visual and auditory systems to probe whether the brain uses entropy or other relevant measures, specially, Fisher information, in aesthetic decisions. We conclude that information measures contribute to these decisions in two ways: first, the absolute quantity of information can modulate aesthetic preferences for certain sensory patterns. However, the preference for volume of information is highly individualized, with information-measures competing with organizing principles, such as rhythm and symmetry. In addition, people tend to be resistant to too much entropy, but not necessarily, high amounts of Fisher information. We show that this resistance may stem in part from the distribution of amount of information in natural sensory stimuli. Second, the measurement of entropic-like quantities over time reveal that they can modulate aesthetic decisions by varying degrees of surprise given temporally integrated expectations. We propose that amount of information underpins complex aesthetic values, possibly informing the brain on the allocation of resources or the situational appropriateness of some cognitive models.

## Introduction

Obtaining information from both society and the environment is essential for the survival of humans and other living beings. From the smallest to the largest creatures, extraction and communication of information helps perform essential functions, such as feeding, mating, or avoiding danger. For example, while exchange of information between ants helps with feeding (Greenwald et al., 2019) whales’ songs help with mating (Suzuki et al., 2006; Smith et al., 2008). Even more prominently, the evolution of sensory systems to help extract key information from the world has been essential to natural selection (Kaas, 1989; Kalmijn, 1989; Endler, 1992; Ellegren, 2008). In a seminal book, Ayres (1997) identifies several types of relevant information. Among the most relevant are those types that serve to reduce uncertainty or were directly relevant to survival. Ayres further classifies the latter type of information as either survival useful or harmful. Frank (2009) shows that the flow of information from the environment to the genome by the process of natural statistically maximize the amount of survival-useful stored knowledge. However, natural selection works at a slow time scale and thus, brains have evolved to get information and make decisions faster.

Over evolution, important tricks were added to the repertoire of animals’ brains to ensure that as much survival-useful information was obtained. For example, animals became experts at using information to adapt to evolving surroundings and overcoming difficulties under rapidly changing conditions (Mobbs et al., 2015). To do so, the brain added knowledge in “improvisation,” with neurons adaptively coding up-to-date information relevant to current surroundings (Duncan, 2001; Grzywacz and Balboa, 2002; Grzywacz and De Juan, 2003). An even more advanced form of adaptability was predictive coding (Srinivasan et al., 1982). With this predictive computation, the brain actively began to extrapolate sensory input, disambiguating present from future information, permitting speedier and optimal reactions to danger (Summerfield et al., 2006; Summerfield and Koechlin, 2008; Mobbs et al., 2015). Furthermore, future-leaning encoding led to better memory retrieval for survival-relevant information (Nairne et al., 2007; Klein et al., 2010). Consequently, given the significance of information, certain circuitries in the brain evolved to process as much information as possible under the constraint of limited neural resources. This was particularly true for the visual system of the brain (Atick and Redlich, 1992; Bialek et al., 1993; Stemmler and Koch, 1999; Balboa and Grzywacz, 2000a,b). And this was pertinent to the evolutionary framework because of the predisposition of the brain to obtain the maximum amount of comprehensible information from natural images (Kaplan and Kaplan, 1989; Heerwagen and Orians, 1995). However, the visual system did not just maximize information, but the kind that was useful for survival in nature (Grzywacz and Balboa, 2002). Brain networks worked best with image statistics associated to natural scenes, which were well-ordered (Field, 1987; Ruderman and Bialek, 1994; Balboa and Grzywacz, 2003). Similarly, the auditory systems of the brain functioned as efficiently as possible with regards to information. The auditory system was matched to characteristics of natural auditory scenes such that naturalistic inputs significantly enhanced the rate of transmitted information in the brain (Attias and Schreiner, 1997, 1998). Moreover, brain employed well-organized encoding mechanisms that expended less computational resources when less information existed in the signal (Overath et al., 2007). Thus, auditory neurons maximized the information of neuronal firing, but considering limits of the energy (Tsubo et al., 2012).

With information being so important and having dedicated neural circuits, one may posit that its amount underlies an aesthetic value. The link to aesthetic value could be direct or through sensory pleasure, with a later conversion to value. A reason for postulating this link is the Processing Fluency theory (Winkielman et al., 2003; Reber et al., 2004). It proposes that the ease of sensory information processing in the brain facilitates aesthetic pleasure. Accordingly, the theory would predict that the quantity of information or a measure related to it, like complexity, underlies an aesthetic value (Aleem et al., 2017, 2019; Correa-Herran et al., 2020). More evidence suggesting that informational volume may underlie an aesthetic value is related to addiction. Most of us have noticed how we are addicted to information through the internet (Widyanto and McMurran, 2004; Ferraro et al., 2006; Byun et al., 2009), social media (Van den Eijnden et al., 2016; Blackwell et al., 2017), and our smartphones (Kwon et al., 2013). Addictive and aesthetic gratifications are linked (Mathis, 2015; Mathis and Han, 2017; Gribkova et al., 2020) through common neural pathways (Adinoff, 2004; Esch and Stefano, 2004; Naqvi and Bechara, 2010), with the connection apparently extending to the realm of information (Chou et al., 1998; Chou and Hsiao, 2000; Song et al., 2004). Addiction to information in the modern world may have a link to the exploration versus exploitation dilemma (Gupta et al., 2006; Dayan and Daw, 2008; Laureiro-Martínez et al., 2015). For example, rodents will change their behavior to exploit or explore more depending on the type of reward (Roesch et al., 2007; Cinotti et al., 2019; Wilson et al., 2021). If the internet makes exploration easy, perhaps information becomes addicting.

That amount of information possibly underlies an aesthetic value is important because values are essential components of decision making (Basten et al., 2010; Glimcher and Fehr, 2013; Ruff and Fehr, 2014). With aesthetic values, decision-making boils down essentially to choice or appraisal. We can decide whether we like this song enough to download, whether we should buy the blue or red shirt, whether that special someone is “our type” enough for dating, or deciding which painting by this up-and-coming artist is our favorite. However, aesthetic choices and appraisal go beyond these categories, extending to everyday life. For example, sociological studies have suggested that all work is like art, with each occupation maintaining a sense of superior production, that is, an occupation aesthetics (Fine, 1992). And sociology and business scholars actively investigate the aesthetics of the workplace (Karlsson, 2012; Sheane, 2012; Sklar and DeLong, 2012). Therefore, aesthetic values are not merely concerned with esoteric or hedonistic aspects of our lives. These values influence many aspects, some essential to survival.

Through review and analysis of the literature, this article primarily asks whether amount of information is an aesthetic value. Our approach to this analysis is proposing questions and then attempting to answer them. Many of the questions are substantial and thus, the literature may not answer them satisfactorily. For each question, we attempt to review the literature thoroughly and then comment on its limitations, specially with an eye toward establishing a link between amount of information and aesthetic values. Here, we do not take the narrower view of aesthetics as only pertaining to art (Hegel, 1998; Danto, 2003; Gaut and Lopes, 2013). Instead, we embrace the broader naturalistic or everyday aesthetics, which allows for influence on appraisal of objects (Saito, 2007; Ratiu, 2013; Mandoki, 2016; Skov and Nadal, 2018). To be able to take this broader view, our review draws from a wide spectrum of disciplines. They include cognitive and computational neuroscience, biology, sociology, economy, business, philosophy, and computer science. The review begins by addressing in Section “What Are Aesthetic Values?” the definition of aesthetic value itself. Then, Section “What Is Information and How Do We Measure It?” addresses the quantification of information. In particular, we will tie the amount of information to surprise, or violated expectation. One of the key concepts in that same section will be Shannon entropy as a measure of information, but inspired by Ayres (1997), we will also address other forms of quantification, specially, Fisher Information. After these background sections on the definitions of information and aesthetic values, Section “Amount of Information As a Possible Aesthetic Value” will review the relevant literature on aesthetic preference based on visual and auditory signals. We will probe whether we can say that their amount of information underlies aesthetic values. We will see that much but not all information has this characteristic, and that its contributions to aesthetic values are highly individual and under social influence.

## What Are Aesthetic Values?

To facilitate reading, we organize this section as follows: The main theoretical concepts are first reviewed (see Sections “A Definition of Values” and “Is Our Definition of Value Compatible With Aesthetic Values?”), with mathematical developments following (see Section “A Brief Mathematical Interlude on Aesthetic Values”). Finally, a recapitulation subsection then provides a brief summary of all these materials.

### A Definition of Values

Values are a central component of decision-making, one of the most important functions of the brain. The goal of decision-making is to choose an action among alternate possibilities. To do so, decision-making invokes a cognitive process based on inputs from the external world, interoceptive information, and assumptions of values of the person making the choice (Simon, 1977; Brown et al., 2011; Aleem et al., 2020). One can study this process from either a psychological/cognitive or normative perspective (Kahneman and Tversky, 2000). The latter often uses expected-value optimization, that is, selecting the alternative with the largest utility, perhaps considering also risk aversion (Fishburn, 2013; Schoemaker, 2013). An alternative that is formally identical, but more commonly used in fields like cognitive science and machine learning is minimization of expected loss (Berger, 2013).

Because expected-value optimization addresses values formally, it is a good starting point for us to consider what they may be in general. The Expected-Utility hypothesis postulates that an agent selects among prospects by calculating expected utility values (Fishburn, 2013; Schoemaker, 2013). These expected values are the sum of predicted payoffs of outcomes weighed by their probabilities. The value function may change depending on the individual. For example, individuals may be risk-averse (Christopoulos et al., 2009; Dohmen et al., 2010; Albert and Duffy, 2012) and the Expected-Utility theory considers this possibility (Hershey and Schoemaker, 1980; Segal and Spivak, 1990; Karni and Schmeidler, 1991). Risk aversion occurs when the value function is concave, that is, it shows diminishing marginal utility (Hanoch and Levy, 1975). Thus, risk-neutral, risk-seeking, and risk-averse people have linear, convex, and concave value functions, respectively.

**FIGURE 1:**
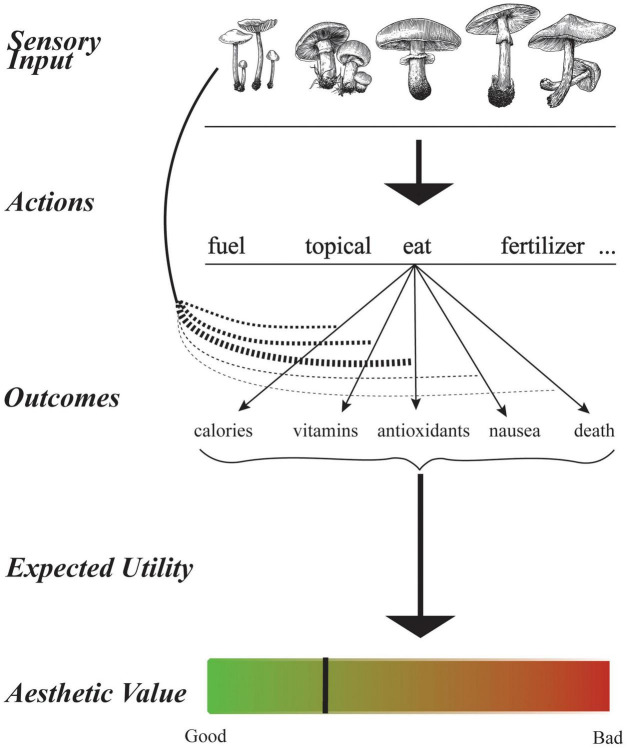
Illustration of proposed relationship between the expected utility hypothesis and aesthetic values. Suppose that a person is seeing a mushroom. The person has many actions from which to choose. The possible actions include, among others, using the mushroom as fuel, topical medicine, food, or fertilizer. When trying to decide what action to take, the person must see to what outcomes an action may lead. For example, the action of eating a mushroom can provide positive or negative utilities such as calories, vitamins, antioxidants, nausea, or death. The Expected Utility Hypothesis proposes to decide what action to take by finding the mean utility of each action and using the optimum. The mean utility considers the probability that an action will lead to each outcome given the sensory input. The probabilities for eating the leftmost mushroom are indicated by the thicknesses of the curved arrows. We propose to equate aesthetic value is a special case of expected utility. For aesthetic values, the only action is evaluation. If the statistics of the sensory input are like those of objects with high expected utility, the aesthetic value of the input is high (Aleem et al., 2019, 2020).

Therefore, if we follow the ideas of expected-value optimization, we should define value as the prediction of payoff or reward (Figure 1). As such, value may be positive or negative. Crucially, the determination of total value depends on the motivation because it signals how often probabilistically the individual will act (Aleem et al., 2020; Grzywacz, 2021). Consequently, value can be calculated as a function of inputs from the external world and interoceptive information, which often also signals the motivation to act (Pessoa, 2009; Brown et al., 2011; Wager and Barrett, 2017).

Some readers may object to a definition of value as a payoff or reward. Instead, these readers may think of value in socio-psychological terms, such as humanitarianism and hedonism, or other like concepts. We grant along with others that this is as deep and complex discussion (Viner, 1925). It allows for other definitions of value that are valid and important. For now, we mention humanistic psychologists have stressed the positive effects of enjoyment as emphasized by Utilitarian philosophers (Veenhoven, 1988). Moreover, a quantitative psychological studied has confirmed factors such as humanitarianism, hedonism, formalism, and dialectics as important utilitarian elements for people (Cheung, 1997). That study suggests that a utilitarian perspective of value may not be incompatible with other socio-psychological points of views.

### Is Our Definition of Value Compatible With Aesthetic Values?

In the context of aesthetic values, this simple definition may be somewhat surprising to most people. It implies that value not only can be positive or negative, but also a non-monotonic function of the underlying variable. Arguably, the misconception about aesthetic values as positive can be “blamed” on some philosophers from ancient Greece until the Enlightenment. Their emphasis is on beauty not ugliness, making us think that positive valence is important, but not negative ones (Gaut and Lopes, 2013; Sartwell, 2017; Aleem et al., 2019). However, negative valence is important, too! The brain must code for things that we both like and do not like. Not only that, but reaching positive and negative values is not a necessarily a linear or monotonic process. Thus, if for example, symmetry is good, it should be a positive value in the sense that the more symmetry a piece has, the better it is. But empirical research shows that this is not always true (Pombo et al., 2021). Instead we should think of aesthetic processing as a two-sided computation, with positive (good) and negative (bad) complements (Brown et al., 2011). The dependence of aesthetic value on, say, variables like amount of symmetry or information, may be a complex, non-linear manifold (Grzywacz, 2021). Thus, aesthetic values do not have to be simple. Notably, non-linear values also play a role in one of the criticisms of the Expected-Utility hypothesis (Kahneman and Tversky, 1979; Aleem et al., 2017; Correa-Herran et al., 2020).

But if this definition of aesthetic values is surprising to some people, others go farther and reject it. A claim is made by some scholars that aesthetic values are socially constructed and thus, they should be discredited as true entities (Bourdieu, 1979; Eagleton, 1983; Eaton, 1995). To these scholars, aesthetic values are significant merely to a social class at a particular historical moment to maintain prominence. We accept and have also argued for the influence of social construction on aesthetic values (Aleem et al., 2019, 2020; Grzywacz, 2021). However, we now contend that this influence is just that, an influence. Moreover, the constraints of the influence of social information are well understood, being most powerful for objects with ambiguous values (Park and Lessig, 1977; Griskevicius et al., 2006; Goldstein et al., 2008).

Instead, we argue that aesthetic values are built-in in specialized brain mechanisms, which are under the influence of evolution, development, and social and environmental learning. To begin with, evidence is now available that genetic predispositions contribute to the sensation of aesthetic chills (Bignardi et al., 2021). Another strong line of evidence comes from development. If aesthetic values are significant merely to maintain social status, then we should not see them early in human development, that is, in babies. However, evidence from many fields shows that babies are born with certain innate preferences. For example, 4-month-old infants have similar aesthetic preferences of facial attractiveness as adults (Samuels et al., 1994). In addition, like adults, 6-month-old infants favor abstract art with contrast and complexity intact (Krentz and Earl, 2013). And although infants do not always have the same visual aesthetic values as adults, babies still have clear preferences (Cacchione et al., 2011; Taylor et al., 2013). Similar results are obtained in the domain of music. Infants just 6 months old pay longer attention to attend to consonant musical intervals than to dissonant ones (Trainor and Heinmiller, 1998). Such evidence suggests that aesthetic values cannot be merely a social construct, but have some biological component.

Neuroimaging and evolutionary studies support the conclusion that biology predisposes humans to have aesthetic values. A major meta-analysis of positive-valence aesthetic-appraisal neuroimaging across four sensory modalities shows the involvement of dedicated centers in the brain. Thus, whether one is appraising visual art, music, gourmet food, or a sophisticated perfume, the same brain areas participate in the aesthetic evaluation (Brown et al., 2011). These kinds of meta-analysis were extended with focuses on faces, visual art, and visual and olfactory aesthetic experiences with similar results (Boccia et al., 2016; Zou et al., 2016; Chuan-Peng et al., 2020; Feng et al., 2021). For good early reviews on the neuroimaging of aesthetic experiences, see the articles by Nadal (2013), and Chatterjee and Vartanian (2014). Brown et al. (2011) gives an excellent summation of the implication of such neuroimaging studies. They write, “one way to naturalize aesthetics is to argue that such a system evolved first for the appraisal of objects of survival advantage, such as food sources, and was later co-opted in humans for the experience of artworks for the satisfaction of social needs.”

This summation begs the question: did aesthetic experiences evolve before humans (O’Hear, 2005)? Evolutionary studies suggest that strategies of animal mate selection underlie the biology of natural aesthetics (Zaidel, 2019). The best example is females of certain species grounding their choice for mating in exaggerated physical characteristics (phenotypes) of males. There has been much debate as to whether these characteristics, such as the peacock’s tail, are purely aesthetic or present an adaptive advantage. A possible reconciliatory perspective is the Fisherian Runaway principle (Fisher, 1915, 1930), which states that certain phenotypes initially disclose significant health-associated information. And females can strongly be claimed to pick the right males, using aesthetic judgment of these phenotypes (Welsch, 2004). This advantage comes at a cost because maintaining these exaggerated phenotypes requires much effort (Zahavi, 1978). However, over time as more mating decisions made based on these phenotypes, they may become uncoupled from their underlying signal, and be chosen for their own sake, leading to a runaway cycle of further exaggeration and uncoupling, sometimes even to a detrimental effect. Darwin himself proposed the existence of an aesthetic sense in animals, introducing the idea of mating selection in his “The Descent of Man” (Darwin, 2008). According to Darwin, an animal’s judgmentis founded on pleasure or attraction, not on an idea or dispassionate analysis (Van Dyck and Darwin, 1882; Welsch, 2004; Darwin, 2008). See Prum (2017) for a historical perspective.

Because we take the position that aesthetic values exist and are not just social constructs, we must ask how our simple definition of value as prediction of payoff or reward compares to others in the literature. A definition like ours has been used before, especially in neuroscience (O’Doherty et al., 2003; Schultz, 2016; Sutton and Barto, 2018) and artificial-intelligence (Alpaydin, 2016; Agrawal et al., 2018; Sutton and Barto, 2018). However, not all definitions are like ours. In Philosophy, for instance, the definitions tend to be broader. For example, (Beardsley, 1979) defined, “the aesthetic value of anything is its capacity to impart-through cognition of it a marked aesthetic character to experience.” This definition broadens the scope of aesthetic value beyond that of our definition to incorporate more than preference. Aesthetic experience also comprehends emotions that range from beauty to wonder, and others (Dufrenne, 1973; Pelowski and Akiba, 2011; Vessel et al., 2013). Therefore, a definition like Beardsley’s is more general than the narrower scope of our definition of aesthetic value, but ours allows for precise quantification and measurement. We feel that we can stick with our definition not because of a criticism of Beardsley, but because we restrict the scope of our work.

A more recent philosophical definition of aesthetic experience is closer to the operational way that we see aesthetic value working (Stecker, 2006). Stecker’s definition maintains that aesthetic experience is, “attending in a discriminating manner to forms, qualities or meaningful features of things, attending to these for their own sake or for the sake of this very experience.” Like Stecker, for us, aesthetic experience is discrimination. It is performed by comparing aesthetic values arising from “forms, qualities or meaningful features of things.” Information, symmetry, and rhythm are examples of these features, each capable of contributing to aesthetic value. However, when Stecker mentions “for their own sake or for the sake of this very experience,” he is raising the issue of disinterest first discussed by Kant (1987). Kant states that the pleasure in the judgment that an object is beautiful generates no interest because the judgment leads to no desire to act (Zangwill, 2021). This statement has been influential (Kemal, 1997), as seen in its use by Stecker. However, the statement is not compatible with our definition of aesthetic values and has been criticized (Guyer, 1978; Nietzsche, 1989; Zangwill, 2021). The main criticism, among others, is that the pleasure in the beautiful can possibly produce desire.

This discussion on Stecker’s ideas leads naturally to an extension of our simple definition of value to the aesthetic realm. Aleem et al. (2019, 2020) proposed that aesthetic value arises from the same brain mechanisms as those predicting reward (Figure 1). To illustrate this point, these authors distinguish two situations: First, when facing an object (for example, an apple), a person may decide to act on it (eat it) if the prediction is of high reward and the motivation is high (hunger). The prediction of reward is based on physical characteristics of the object (for example, shape, color, and absence of smudges in the apple). Thus, if the object is beautiful and the motivation is high, the person will act (eat the apple). Second, when facing a painting of the object (of the apple) or a painting that has the visual statistics of the object, the stimulus will act on the same brain areas. They, in turn, will respond with a prediction of reward. However, the person cannot eat the object (apple) in the painting and thus, has Kantian disinterest. The bottom-line is that, according to Aleem et al. (2019, 2020), beauty can lead to interest or disinterest depending on the situation. Different situations do not employ different brain mechanisms. Hence, from an everyday-aesthetics perspective, all-around disinterest would not be justified. But as stated above, Kant worked at a time when art was the realm of aesthetics and it referred only to beauty, not to ugliness. Consequently, Kant (and Stecker) was justified under this narrower definition of aesthetics to speak of disinterestedness.

### A Brief Mathematical Interlude on Aesthetic Values

As we have pointed out in Section “Is Our Definition of Value Compatible With Aesthetic Values?”, our definition of value “allows for precise quantification and measurement.” In this section, we briefly show how some authors have performed this quantification. In this review, we adapt the aesthetic-value notation used by Grzywacz (2021).

The goal of values is to help select the best option among many candidate actions. The Expected-Utility hypothesis proposes that an agent selects between the candidates by comparing the expected (mean) values. To show how to calculate expected values, we begin by denoting the *k^th^* possible action by *a_k_*. Each action may lead probabilistically to different possible outcomes, which we represent by *o*_*i*_, 1 ≤ *i* ≤ *M*.

The probability that the Action *a_k_* leads to Outcome *o_i_* is *p*_*k,i*_, a function of the sensory signals $\vec{u}$, with $\vec{w}$ being the parameters of the function. The sensory signals, $\vec{u}\left(t\right)$, are represented as a vector, with the components being the variables that characterize the external world. For example, components of the stimulus vector may be the amount of information, color, or average rhythm. The vector of parameters, $\vec{w}$, may not have the same size as $\vec{u}$.

The follow example illustrates how *p*_*k,i*_ works. What is the probability that if I eat this apple (action) with 7-cm diameter and this particular tone of red (sensory stimuli), I get 19 g of sugar (reward)? Thus, each possible outcome *o_i_* of the action leads to a reward, whose value (predicted reward) is v*(*o*_*i*_).

The probably *p*_*k,i*_ and the value of the outcomes v*(*o*_*i*_) allows to compute the expected utility as


(1) $$U\left(a_{k},\vec{u}\left(t\right):\vec{w}\left(t\right)\right)=\sum_{i=1}^{M}p_{k,i}\left(\vec{u}\left(t\right):\vec{w}\left(t\right)\right)v^{*}\left(o_{i}\right)$$

where the colons in Equation 1 designate parameters and thus, for example, $p_{k,i}\left(\vec{u}\left(t\right):\vec{w}\left(t\right)\right)$ indicates that *p*_*k,i*_ has $\vec{u}$ as variables and $\vec{w}$ as parameters. The reason $\vec{w}$ varies with time is that learning may operate to optimize the parameters – (Sutton and Barto, 2018; Aleem et al., 2020; Grzywacz, 2021). Different models of learning have been proposed, with the most common being in the form of differential equations (Rescorla and Wagner, 1972; Pearce and Hall, 1980; Sutton and Barto, 2018) or Bayesian updating (Strens, 2000; Ghavamzadeh et al., 2015; Vlassis et al., 2021). All these models learn parameters by comparing the value *v*(*t*), that is, the prediction of reward, with the actual reward.

According to the discussion in Section “A Definition of Values,” we define value function, μ_*G*_, as the expected utility in Equation 1:


(2) $$U_{G}\left(a_{k},\vec{u}\left(t\right):\vec{w}\left(t\right)\right)=\mu_{G}\left(a_{k},\vec{u}\left(t\right):\vec{w}\left(t\right)\right)$$

where the subindex *G* indicates that we are talking about value in general, not just aesthetic (Figure 1). Therefore, the value function is the expected utility with an action and the sensory stimulus given.

For aesthetic experiences, the only action is the evaluation itself. This is not a trivial point because we could consider actions like continue looking at an object of interest, touching it, buying it, or going to the museum. What must consider what the primary action caused by aesthetic value is. Is it evaluation, as we suggest, which then leads to other actions, or are aesthetic values leading directly to other actions, bypassing evaluation as a first step? More research will be needed to settle these questions. However, we take the evaluation-first approach because it both makes the theory simpler and leads to similar results. Thus, in our mathematical development, *a_k_* is not a variable. We should then replace Equation 2 with


(3) $$U_{A}\left(\vec{u}\left(t\right):\vec{w}\left(t\right)\right)=\mu_{A}\left(\vec{u}\left(t\right):\vec{w}\left(t\right)\right)$$

where the subindex *A* indicates that we are talking about aesthetic value (Figure 1). The article by Grzywacz (2021) presents many examples of μ_*A*_, some simple and linear, and some complex and non-linear.

The only thing missing to calculate the aesthetic value is the insertion of motivation into the mix. This is done as follows:


(4) $$v\left(t\right)=m\left(t\right)\mu_{A}\left(\vec{u}\left(t\right):\vec{w}\left(t\right)\right)$$

where 0 ≤ *m* ≤ 1 is the motivation function (interpreted as the probability of acting). Because the motivation function *m* causes *v* ≤ μ_*A*_, we must interpret μ_*A*_ as the fully motivated value.

### Recapitulation of Section “What Are Aesthetic Values”

This section grounds aesthetics as values, indifferent from other values that enable decision-making. This grounding allows us to take ideas from well-established theories, such as the Expected Value Hypothesis, and apply it to the aesthetic domain. Thus, extending aesthetic values to both positive and negative realms, as required by decision-making models, may be necessary to understand aesthetic phenomena. We then extend this approach by looking back to the roots of value itself, exploring the role of value as a component of appraisal mechanisms underlying survival. This extension shows us that aesthetic values are not purely social constructs. Instead, aesthetic values have roots in essential human behavior through evolution, as supported by genetic and developmental evidence. To quantify aesthetic values, we look at different philosophical and technical definitions. From this look, we come to a definition fit for our scope, namely, that aesthetic value has a positive relation to expected reward. This definition allows us to work with useful models in neuroscience and economics, and apply them to aesthetics.

## What Is Information and How Do We Measure It?

The organization of this section is like that in Section “What Are Aesthetic Values?”. The main theoretical concepts related to amount of information (see Sections “Information Versus Amount of Information,” “The Nature of Information,” and “Amount of Information”) are followed by the corresponding mathematical developments (see Section “A Brief Mathematical Interlude on Amount of Information”). Finally, a recapitulation subsection then provides a brief summary of all these materials.

### Information Versus Amount of Information

In this article, we review and discuss the literature to probe whether the amount information, not information itself, underpins aesthetic values. This may seem strange to some who may argue that the semantic meaning of information is what moves us. For example, many people cannot see Michelangelo’s *La Pietà* (Pope-Hennessy, 1970) without experiencing meaningful feelings. They involve the blend agony of a mother over the death of her son, and her tenderness and heroic resignation. Tenderness and heroism are values for most of us. Hence, one can write books and articles about these values in the aesthetic domain (Feldman, 2002; Brattico et al., 2013; Starr, 2013). However, this article asks whether amount of information in itself supports aesthetic values, not whether information or specific messages are values. In a sense, all sensory signals carry information. Consequently, asking whether it has values in itself is meaningless because it would be the same as saying that all sensory stimuli are equally valuable. However, amount of information may support a value because when the quantity is high, it can alert the brain that it may find valuable messages in the incoming information. If one reasonably assumes that the chance of finding such valuable messages has a positive correlation with the amount of information, thus, this amount becomes a prediction of reward. With more information, the brain should thus devote more resources to process the incoming sensory signals. Thus, the amount of information may be valuable as a type of attentional mechanism, as proposed previously by several authors (Stolnitz, 1960; Nanay, 2015, 2016; Fazekas, 2016).

### The Nature of Information

The concept of information is so important that many books and articles have been written on the subject (Lombardi, 2004; Floridi, 2010; Israel and Perry, 2012; Adami, 2016; Janich, 2018). This literature often points out that although most of us has an intuitive feeling about what information is, its connection to knowledge is not always transparent. This connection is clarified in one of three ways: (1) information is the resolution of uncertainty; (2) information is what lets us make predictions with reasonably good accuracy; (3) information is data that empower decision making. These are not independent. For example, the resolution of uncertainty cannot happen without making predictions from data. An important question thus becomes, what should we be predicting? To this, Adami (2016) elaborates, “Well, in general, when we make predictions, they are about a system that we do not already know. In other words, another system… I have to specify this ‘other system’ as precisely as I can. I have to specify, in particular, which states the system can take on … information … [depends on] … the number of unknown states [of this other system].”

The concept of information has diverse significances in different backgrounds (Lombardi, 2004; Floridi, 2010; Israel and Perry, 2012). At the simplest level, information has become tantamount to communication, data, education, knowledge, and entropy, among other concepts. This multiplicity of meanings may occur because if the same event occurs in two different backgrounds, where dissimilar constraints exist, it might yield two different information contents (Israel and Perry, 2012). Lombardi (2004) went deeper into problem and identified three major approaches to understand information. She called them the semantic, syntactic, and interaction-information approaches.

Arguably, the most appealing approach for aesthetic experiences comes from the semantic theory of information (Dretske, 1981). This theory emphasizes the necessity of theories of information to express something about its content. Semantic aspects of information have undeniable effects on aesthetic experiences (Locher et al., 2008; Kirk et al., 2009; Krishna et al., 2010; Chatterjee and Vartanian, 2014). However, despite efforts by Dretske (1981; Lombardi, 2004), no complete method is available to measure the amount of semantic information, although some recent significant progress in this direction is worth mentioning (Kolchinsky and Wolpert, 2018). A similar limitation applies to the interaction-information interpretation of scientific observation developed by Kosso (1989) and emphasized by Lombardi (2004). As the name of the interpretation implies, this approach defines information through the observable interactions between entities that ultimately allow us to make scientific inferences. This is insightful, but does not lead to a concrete method to measure the amount of information.

The only approach among those highlighted by Lombardi (2004) that can lead to measurement of the amount of information is the syntactic approach of Cover and Thomas (2006). In this information-science perspective, the characterization of information has nothing to do with communication, transmission, and reception of messages. And information has nothing to do with the knowledge of an event acquired by observing another. Random variables and their correlations are the substances of this approach to information. Thus, unfortunately, this approach lacks the elementary insight of information augmenting the recipient’s knowledge. However, on the other side, the approach transforms information into a syntactic idea, allowing application to multiple areas of study.

### Amount of Information

#### Shannon Entropy

The most common way to apply the syntactic approach to information mentioned in the previous section has been through principles of Information Theory (Shannon, 1948; MacKay, 2003; Cover and Thomas, 2006). Multiple examples of such applications exist in the area of aesthetic values in both the visual (Arnheim, 1974; Stamps<suffix>III.</suffix>, 2002; Rigau et al., 2007, 2008; Aleem et al., 2017; Correa-Herran et al., 2020) and auditory (Meyer, 1957; Knopoff and Hutchinson, 1981; Temperley, 2007; Rohrmeier and Koelsch, 2012; Agres et al., 2013; Miles et al., 2017, 2021a; Delplanque et al., 2019) domains.

**FIGURE 2:**
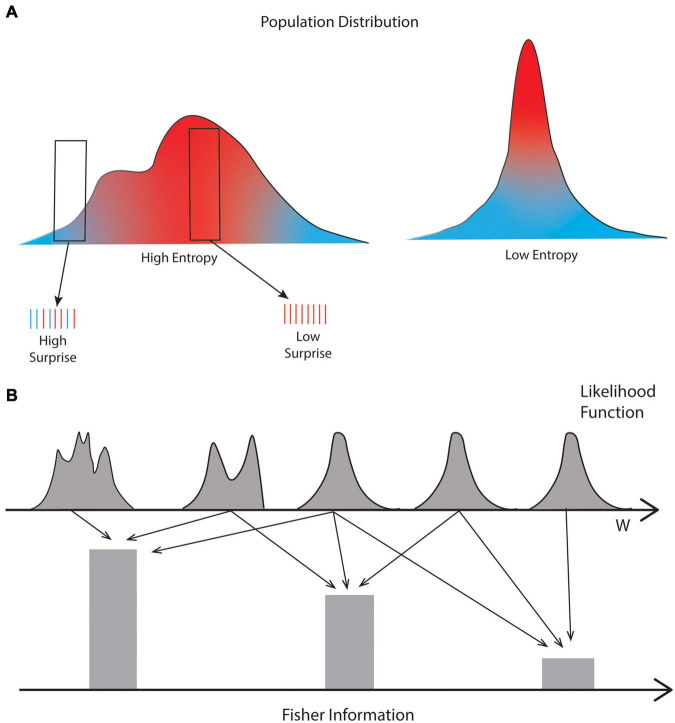
Illustration of the differences between Shannon entropy, amount of surprise, and Fisher information. **(A)** Shannon entropy captures the spread of the statistics of the population (red and blue curves), such that with more spread, we have more Entropy. In turn, amount of surprise captures how unusual a sample is. For example, the left sample in the figure has elements that are relatively common (red vertical lines) and highly unusual (blue lines), that is, in the fringe of the distribution. By contrast, the right sample only has common elements. Therefore, the left and right samples have high and low amount of surprise, respectively. **(B)** Fisher Information captures how much information the likelihood function has about its parameter (w). For range of parameters causing great changes of the likelihood function, the Fisher Information is high. The opposite occurs for a range of parameters where the likelihood function is relatively constant. Consequently, while Shannon entropy and Amount of Surprise capture properties of the sensory inputs, Fisher Information captures properties of parameters of cognitive models in the brain.

The core of the application of Information Theory to the measurement of the amount of information is Shannon entropy. This statistical measure is called entropy because of its similarity to equations of entropy in statistical mechanics (Ellis, 2006). In Physics, entropy is commonly associated with randomness and a state of disorder. Relatedly, Shannon entropy measures statistical uncertainty. To be precise, the Shannon entropy of a random variable is the mean amount of uncertainty in the potential outcomes of the variable (Figure 2). If so, entropy captures the available information, because Shannon proposed that the role of information is to reduce uncertainty. As such, the entropy-based implementation of amount of information is compatible with the Ayres’s uncertainty-reducing type discussed in the *Introduction* (Ayres, 1997).

To explain in more detail how to use Shannon entropy in the visual domain, we follow the presentation of Aleem et al. (2017). For a similar presentation in the auditory domain, see the work of Miles et al. (2017, 2021a). They wanted to measure complexity in visual images, especially in paintings. In many of the past studies, visual complexity was defined in simple terms, such as the number of features or a perceptual scale (Berlyne, 1973; Aitken, 1974; Nicki and Moss, 1975; Imamoglu, 2000). Aleem et al. (2017) wanted to define complexity more rigorously and in a way that would be consistent across studies. They then realized out that complexity and entropy had a strong connection.

To apply Shannon entropy to an image, one must define random variables applicable to it. Aleem et al. (2017) chose pixel intensities as such variables. First, they considered the probability distribution of intensities in each of their images and measured entropy from this distribution. However, entropy was an extensive variable (Tsallis, 1988, 2009), that is, it differed with the quantity of material (pixels, in our case). Aleem et al. (2017) thus proceeded to normalize the entropy by dividing it by its largest possible value given any arbitrary image of the same size. This largest entropy came from images for which the pixels had intensities randomly picked from all possible values. They called this normalized entropy the Complexity of Order 1. Second, Aleem et al. (2017) realized that a limitation of Complexity of Order 1 was that it did not account for modifications in entropy due to spatial organization. If one scrambled the pixels in an image, it looked more complex, but the Complexity of Order 1 stayed the same. To incorporate the spatial modification of complexity, Aleem et al. (2017) performed arbitrary isometric (that is, distance preserving) transformations of the image. Then, they asked if two juxtaposed pixels predict the intensities of each other after the transformations. This method was also normalized, but this time using conditional probabilities of the intensity of a pixel predicting the intensity of another after the transformation. Because they used two pixels, they called the emerging quantity Complexity of Order 2.

#### Degree of Surprise Given Temporally Integrated Expectations

Surprise has been an important concept in aesthetics research, specially in the realm of music (Huron, 2008; Meyer, 2008). In that realm, surprise has been used with two different meanings. First, surprise appears as an emotion (Barto et al., 2013). This is the emotion that ensues from a discrepancy between an expectation and an observation. In the music-cognition literature, this emotion is even more complex, with surprise often having a negative connotation (Huron, 2008). This happens because surprise or violation of expectation is equivalent to “something is wrong here.” Thus, part of the pleasure in music is thought to be due to the discharge from the tension prompted by surprise. Second, surprise appears as a quantity (Miles et al., 2017, 2021a). Basically, a surprise is a violation of expectation and thus, one can define it precisely in terms of an underlying probability distribution (Egermann et al., 2013). In Information Theory, the amount of surprise is minus the logarithm of the probability of an event and thus, the smaller its probability, the larger is this amount. In the rest of this article, we will use “amount of surprise” to refer to this Information Theory quantity or averages of it instead of just “surprise,” which will refer to the emotion.

In this article, we will consider amount of surprise over temporally integrated expectations. As we will see in Section “Shannon-Entropy Measures,” Shannon Entropy is simply amount of surprise averaged over the population. However, when we discuss average amount of surprise in this article, we do not mean Entropy. We mean amount of surprise averaged over the members of a sample. The surprise of each member is always relative to the population not to the statistics of the sample (Figure 2). In general, one cannot talk about the entropy of a sample because it may only have one member. An example of a sample used in the literature is the set of chords in a section of a song (Miles et al., 2021a,b). In this case, the population probabilities could arise from “all” songs in a year or integrated over several years. Because amount of surprise is the property of a sample, one can say that it carries large amounts of unusual information when the amount of surprise is high. In aesthetics, the probabilities underlying amount of surprise are often instantaneous, for example, the distribution of intensities in a painting. However, in the case of amount of surprise over temporally integrated expectations, one instead uses statistics that accumulate over time and thus, possibly changes continuously. For example, when Miles et al. (2021a) studied surprise in songs released in, say, 1980, they compared them to songs released for many years before that time point. Because they did the same for 1990, the underlying statistics could be different. What was surprise in 1980 might not be surprise in 1990. Thus, these authors still used entropy to measure amount of surprise but did so in terms of temporally integrated statistics.

These examples highlighted that depending on the variables that one chose, one could emerge with different types of entropy. Our examples involved intensities and positions, but others could involve colors, textures, shapes, or other visual properties. The same applied to the auditory domain. For example, Miles et al. (2017, 2021a) used harmony as their variable in the analysis of surprise in popular music. In contrast, Delplanque et al. (2019) used pure tones as their variable. And one can conceive more complex distribution involving a multidimensional auditory space, such as combining harmonies and time intervals (rhythm), to obtain measures like the Complexity of Order 2 of Aleem et al. (2017).

#### Fisher Information

The measures of information above address Ayres’s uncertainty-reducing type of information, but not necessarily his survival-relevant type (Ayres, 1997). This is important because Shannon entropy measures all available information regardless whether it is useful. However, the visual system does not just maximize processed information, but the kind that is useful for survival in nature (Grzywacz and Balboa, 2002). Brain networks work best with natural statistics regardless if they are visual (Field, 1987; Ruderman and Bialek, 1994; Balboa and Grzywacz, 2003) or auditory (Attias and Schreiner, 1997, 1998). Furthermore, the brain may work best in tasks with naturalistic statistical structure (Botvinick et al., 2015, 2019, 2020). Hence, we ought to consider an information measure focused on natural-survival statistics rather than on all information.

We propose that the best alternative in terms of survival-relevant amount of information is Fisher Information (Rissanen, 1996; Frieden and Gatenby, 2007; Ly et al., 2017). The starting point for this proposal is the work of Frank (2009). He shows that natural selection maximizes Fisher Information. From this result, we have asked whether Fisher Information captures valuable information better than does Shannon entropy. This is not an easy question to answer because Fisher Information and Shannon entropy have important differences. The main difference, as we will see next, is that Fisher Information but not Shannon entropy is model dependent. We propose that a possible function of Fisher Information is to help the brain decide on the resources necessary to establish good parameters for its cognitive models. As the social or environmental situation changes, the parameters of some perceptual brain models may momentarily not be ideal. To correct this parametric deficiency, the brain may trigger learning (Sutton and Barto, 2018; Aleem et al., 2020; Grzywacz, 2021) or sensory adaptation (Duncan, 2001; Grzywacz and Balboa, 2002; Grzywacz and De Juan, 2003). Fisher Information helps in determining how much sensory data the brain must integrate during the learning or adaptation process.

Formally, Fisher Information is the mean amount of information that discernable random variables, sensory and interoceptive signals in our case, convey about parameters of a distribution, the likelihood function (Myung, 2003), of the random variables (Figure 2). Thus, Fisher Information models, and accordingly, our models of cognition, must be probabilistic. Fortunately, many models of visual and auditory cognition have been appropriately Bayesian for over 25 years (Knill and Richards, 1996; Kersten et al., 2004; Knill and Pouget, 2004; Colombo and Seriès, 2012; Cusimano et al., 2018). Any of these Bayesian models are appropriate for the applications of Fisher Information. However, in this article, we will focus on examples of learning models of aesthetic values. Many models for learning and adaptation in the brain are Bayesian. In the learning domain, the area most appropriate for aesthetic values is model-based Bayesian reinforcement learning (Strens, 2000; Ghavamzadeh et al., 2015; Vlassis et al., 2021). Such Bayesian models of learning typically take the form of Kalman filtering (Sutton, 1992; Dayan and Kakade, 2000; Kakade and Dayan, 2002). Similarly, Kalman filtering is also the basis for Bayesian models of sensory adaptation (Grzywacz and De Juan, 2003; Barraza and Grzywacz, 2008). The commonality of mechanisms for the learning and adaptation processes is not surprising. The study of both processes begins by building the probability function of the parameters of a brain model given the history of the incoming sensory stimuli. When one applies Bayes’ Theorem to this probability function, the result is the typical multiplication of the likelihood function by the prior distribution. In this case, the condition of the likelihood function contains the parameter of the brain model as required by the equations of Fisher Information.

A related concept is Observed Fisher Information (Efron and Hinkley, 1978; Palmgren, 1981). While the Fisher Information is information averaged over all possible values of the discernable random variables, Observed Fisher Information is information obtained for just the last measurement. Because the brain has access to each measurement, the process to estimate Fisher information may be through a sequential sampling based on the Observed Fisher Information (Grambsch, 1983). Such a sequential procedure would potentially have a benefit. The Observed Fisher Information could provide a rapid test of whether the current parameters of the likelihood function are good. Thus, the Observed Fisher Information would help the brain determine whether what it currently believes is appropriate for the present situation. With some exceptions (Efron and Hinkley, 1978), this information would not normally be as good as the full Fisher Information. However, the speed of the estimation of the Observed Fisher Information may still make it worthwhile as a survival type of information. This speed could also be useful for rapidly changing artistic stimuli, such as movies or dance.

### A Brief Mathematical Interlude on Amount of Information

The goal of this section is to define amount of information as a possible basis for aesthetic values. We thus must ensure that the definitions are compatible with the notations in Section “A Brief Mathematical Interlude on Aesthetic Values”. In this section, we begin with concepts around Shannon entropy (see Section “Shannon-Entropy Measures”) and conclude with ideas related to Fisher Information (see Section “Fisher-Information Measures”).

#### Shannon-Entropy Measures

As in Section “Amount of Information,” we illustrate the use of Shannon entropy for aesthetic values in the visual domain, following the presentation of Aleem et al. (2017). They began by measuring in Image *Q*(*t*) the probability $P_{Q\left(t\right)}^{\left(1\right)}\left(l\right)$ of Intensity *l*. The amount of surprise due to this intensity is $-log_{2}\left(P_{Q\left(t\right)}^{\left(1\right)}\left(l\right)\right)$ and its expectation is the Shannon entropy of Order 1 (Figure 2):


(5) $$H_{1}\left(Q\left(t\right)\right)=-\sum_{l=0}^{I^{*}}P_{Q\left(t\right)}^{\left(1\right)}\left(l\right)log_{2}\left(P_{Q\left(t\right)}^{\left(1\right)}\left(l\right)\right)$$

where I* was the maximally possible intensity [255 for Aleem et al. (2020)]. To create an index of complexity out of this entropy, they divided *H*_1_(*Q*) it by its largest possible value given any arbitrary image. The result was the Complexity of Order 1:


(6) $$u_{C_{1}}\left(Q\left(t\right)\right)=-\sum_{l=0}^{I^{*}}P_{Q\left(t\right)}^{\left(1\right)}\left(l\right)log_{I^{*}+1}\left(P_{Q\left(t\right)}^{\left(1\right)}\left(l\right)\right)$$

where *u*_*C*_1__ was the variable used in value function as in Equation 3. Because of the division of *H*_1_ (*Q*) by its largest possible value, 0 ≤ *u*_*C*_1__ (*Q*(*t*)) ≤ 1, with 0 happening for single-tone images (that is, the simplest ones) and 1 happening for images whose intensities are spread homogeneously and randomly through all possible values.

Aleem et al. (2020) also defined Complexity of Order 2 by considering complexity due to both intensities and spatial organization. The generalized the procedure used for Equation 5 by first measuring in Image *Q*(*t*) the probability $P_{Q\left(t\right)}^{\left(2\right)}\left(l_{2}|l_{1},T\right)$ that a pixel with Intensity l_1_ was juxtaposed with a pixel with Intensity l_2_ after the isometric transformation *T*. From this measurement, they defined Shannon entropy of Order 2 for Image *Q*(*t*) and Transformation *T* as


(7) $$\begin{array}{c} H_{2}\left(Q\left(t\right),T\right)=-\sum_{l_{1}=0}^{I^{*}}P_{Q\left(t\right)}^{\left(1\right)}\left(l_{1}\right)\sum_{l_{2}=0}^{I^{*}}P_{Q\left(t\right)}^{\left(2\right)}\left(l_{2}|l_{1},T\right)\\ log_{2}\left(P_{Q\left(t\right)}^{\left(2\right)}\left(l_{2}|l_{1},T\right)\right) \end{array}$$

Finally, they got the Complexity of Order 2, *u*_*C*_2__(*Q*(*t*), *T*), by again dividing by the maximally possible value of *H*_2_(*Q*(*t*), *T*). Again, because of this division, 0 ≤ *u*_*C*_2__(*Q*(*t*), *T*) ≤ 1. In the figure reporting Complexity of Order 2 in this article, we follow Aleem et al. (2020) and obtain the mean over all possible *T*′s.

The examples in Equations 5–7 are just to show the path for further calculations using Shannon entropy. Many other similar examples are possible, such as chromatic and visual-texture complexity.

Grzywacz (2021) proposed a value function for *u*_*C*_1__ and *u*_*C*_2__ based on experimental results in the literature and the work of Aleem et al. (2020). In the notation of this paper (Equation 4), this value function is


(8) $$\mu_{ale}\left(u_{C_{i}}:\vec{w}\right)=-w_{1}\theta \left(w_{2},w_{3}\right)+w_{1}e^{-\frac{{\left(u_{C_{i}}-w_{2}\right)}^{2}}{2w_{3}^{2}}}$$

where the subscript *ale* stands for Aleem et al. (2020) and θ(*w*_2_, *w*_3_) is a constant ensuring that the integral of over the range of *u*_*C_i_*_ is zero. The Gaussian in Equation 8 helps capture the inverted-U-shape dependence of aesthetic preference on complexity as we will explain in Section “Amount of Information As a Possible Aesthetic Value.”

#### Fisher-Information Measures

Different from Shannon entropy, the application of Fisher Information to model aesthetic values is in its infancy. However, Section “Amount of Information” describes the principles of this application (Figure 2), whose basis is Kalman filtering models of reinforcement learning (Sutton, 1992; Dayan and Kakade, 2000; Kakade and Dayan, 2002). One can express these models as the multiplication of the likelihood and prior functions. As mentioned in Section “Amount of Information”, the likelihood function is the one that is key for Fisher Information.

When talking about aesthetic values, the likelihood function underlying the Fisher Information must be related to Equation 4. The inputs in that equation are *m*(*t*) and $\vec{u}\left(t\right)$, and the output is the value. During learning, this value is compared to the actual reward, *r*(*t*), with the error being reduced over time. Consequently, we have three inputs, *m*(*t*), $\vec{u}\left(t\right)$, and *r*(*t*), providing Fisher Information on the parameters $\vec{w}\left(t\right)$, making the likelihood function $P_{L}\left(\vec{u}\left(t\right),m\left(t\right),r\left(t\right)|\vec{w}\left(t\right)\right)$. In most Kalman models of reinforcement learning, this probability is a decreasing function of the error, that is,


(9) $$\begin{array}{c} P_{L}\left(r\left(t\right),m\left(t\right),\vec{u}\left(t\right)|\vec{w}\left(t\right)\right)\\ =P_{L}\left(r\left(t\right)-m\left(t\right)\mu_{A}\left(\vec{u}\left(t\right):\vec{w}\left(t\right)\right)\right) \end{array}$$

Not only that, but in most Kalman models of reinforcement learning, the function *P_L_* is Gaussian (Gershman, 2015; Foley and Marjoram, 2017; Piray and Daw, 2020).

Because $\vec{w}\left(t\right)$ can include many parameters, Fisher Information will not be a single number but a matrix. The Fisher Information Matrix is defined as the covariance of the partial derivatives of the log-likelihood function by its various parameters. However, here, we will assume well-established regularity conditions (Lehmann and Casella, 2006; Schervish, 2012) to simplify the Fisher Information Matrix to


(10) $$\begin{array}{c} {\left[ℱ\left(\vec{w}\right)\right]}_{i,j}=-\int_{\vec{u},m,r}P_{L}\left(r,m,\vec{u}|\vec{w}\right)\frac{\partial^{2}}{\partial w_{i}\partial w_{j}}\\ logP_{L}\left(r,m,\vec{u}|\vec{w}\right) \end{array}$$

We assume the regularity conditions because value functions (for example, Equation 8) and likelihood functions (for example, Equation 9 as a Gaussian) typically used in aesthetic-value research obey such conditions (Grzywacz, 2021).

As discussed in Section “Amount of Information,” a related measure of importance is the Observed Fisher Information. It captures an easier-to-measure, though rougher, estimation of the information that the input variables provide about the parameters $\vec{w}$. This estimation is easier to measure because it bypasses the integral in Equation 10. The estimation uses part of the integrand in that equation and is


(11) $${\left[{ℱ}_{O}\left(\vec{w}\right)\right]}_{i,j}=-\frac{\partial^{2}}{\partial w_{i}\partial w_{j}}logP_{L}\left(r,m,\vec{u}|\vec{w}\right)$$

To summarize, the application of Fisher Information to aesthetic-value research would proceed by combining either Equation 10 or Equation 11 with a suitable likelihood function in the form of Equation 9 and an appropriate value function (for example, Equation 8). These calculations would provide a Fisher Information Matrix (or its observed version) at all moments of the learning process. What is the interpretation of this matrix and why is it called information? Equation 10 shows that the matrix is the negative expected Hessian of the log-likelihood function. Therefore, the Fisher Information Matrix is a curvature matrix of the log-likelihood. What is the interpretation of the various entries of this matrix? From the explanation before Equation 10, the diagonal elements of the matrix are the variances of the first partial derivatives of the log-likelihood function by its various parameters. If one of these variances is near zero, then the partial derivative by this parameter is essentially constant in the range of important values of ($r,m,\vec{u}$). Hence, these values *carry little information* about the parameter. The oppositive is true when a diagonal entry is large. What do the off-diagonal elements represent? If one of these elements is large, it indicates a high degree of covariance between partial derivatives of the log-likelihood function by two different parameters. Consequently, large off-diagonal terms mean that $\left(r,m,\vec{u}\right)$ carry high amounts of information about parametric co-dependence. In contrast, when an off-diagonal term is near zero, the corresponding parameters do not co-depend significantly in the range of important values of ($r,m,\vec{u}$). Similar arguments would apply to the diagonal and off-diagonal entries of the observed matrix Equation 11.

### Recapitulation of Section “What Is Information and How Do We Measure It?”

To analyze the possible connection between amount of information and aesthetic values, we first dive into the nature of information itself. We probe through many formal definitions of information, and settle on one, the syntactic approach, which allows us to make quantitative approximations. The most influential quantitative exploration of information has been Shannon’s Entropy. It can provide a measure of the amount of information available in the sensory input. This definition of information has been widely useful across many scientific disciplines, including aesthetics. As an example, we describe a case-study of how visual complexity (amount of information) of an image can be measured through an analog of Shannon entropy. Furthermore, an extension of Shannon entropy, known as surprise, has been influential in music research. In simple terms, surprise increases as the likelihood of a musical event decreases. However, these definitions of amount of information and surprise are indifferent to the relevance to survival of parts of that information. To overcome this difficulty, we propose to use a definition where the amount of information is a quantity contingent on the observer, in our case, the brain. This viewpoint brings us to a measure of information called Fisher information. Fisher’s definition differs from Shannon’s in that Fisher views information through a likelihood model. Thus, only information that helps improve the parameters of the model is useful information. While scholars have not yet studied Fisher Information extensively in cognitive science, evidence suggests that natural selection aims to increase this type of information.

## Amount of Information as a Possible Aesthetic Value

We organize this section in a fashion like in Sections “What Are Aesthetic Values?” and “What Is Information and How Do We Measure It?”. In the first subsections (see Sections “When More Information Increases Aesthetic Values,” “When More Information Reduces Aesthetic Values,” and “Amount of Surprise Over Temporally Integrated Expectations”), we describe the dependence of aesthetic values on the amount of information. Then, in Subsections “Why Aesthetic Values Show an Inverted-U-Shape Dependence on Amount of Information” and “Fisher Information: Appraisal of Brain Models and Inverted-U-Shape Behavior,” we address why the dependence looks like it does. Finally, again, a recapitulation subsection then provides a brief summary of all these materials.

### When More Information Increases Aesthetic Values

The best starting point to review amount of information as possibly supporting aesthetic values is to consider its connection to complexity. As seen in Sections “Amount of Information” and “A Brief Mathematical Interlude on Amount of Information,” complexity has a formal connection to Shannon entropy. Moreover, complexity has historically been thought to be one of the factors underlying aesthetic experiences (Fechner, 1876; Gilbert and Kuhn, 1941; Rist, 1967).

The link between information and aesthetic preference is solidified through the finding that in many studies, aesthetic preferences are increased with complexity. This increase certainly occurs in the visual domain for abstract art (Osborne and Farley, 1970; Mayer and Landwehr, 2014), and the perception of visual textures (Bies et al., 2016; Friedenberg and Liby, 2016) and snowflakes (Adkins and Norman, 2016). These studies computed complexity using variables like density, fractal dimensionality, and the size of the ZIP compressed file. The increase of aesthetic preferences with complexity also occurred with music, using chords as the variable (Miles et al., 2017, 2021a). Finally, in the arts, the effect happened with dance, as measured by image velocities (Orlandi et al., 2020). In the consumer domain, a complex interface design for smartwatches is more likely to cause emotional arousal and valence than simple interface (Wang and Hsu, 2020).

**FIGURE 3:**
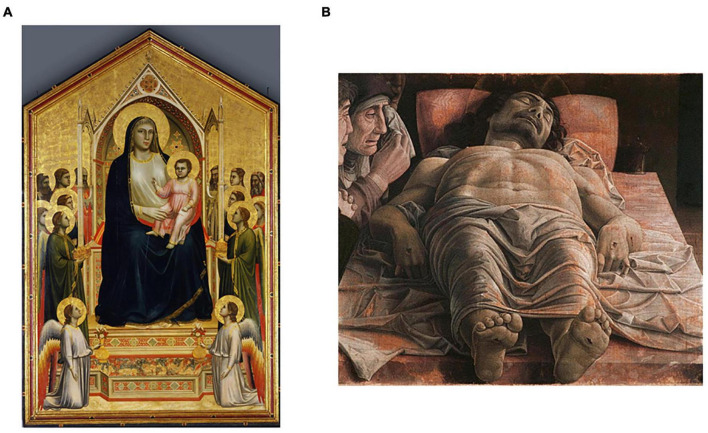
The amount of information in paintings increased through deliberate effort from the middle ages to the renaissance. **(A)** Madonna and Child, Giotto di Bondone, 1320. **(B)** The Lamentation Over the Dead Christ, Andrea Mantegna, 1490. These paintings were obtained from public domain sites in the internet with links: https://upload.wikimedia.org/wikipedia/commons/e/e3/Giotto_di_Bondone_090.jpg and https://commons.wikimedia.org/wiki/File:Andrea_Mantegna_-_The_Lamentation_over_the_Dead_Christ_-_WGA13981.jpg.

More evidence for amount of information supporting aesthetic values emerges from a scientific analysis of art history. Leonardo da Vinci (Jones, 2012; Perloff, 2013) and Michelangelo (Eknoyan, 2000; Suk and Tamargo, 2010) went to the extreme of dissecting corpses to increase the realism and thus the complexity of their art. A discernable increase in the amount of complexity in paintings during the Renaissance ensued, with striking results (Figure 3). New concepts were continuously discovered or rediscovered, and introduced in the work of artists (Alberti, 2013). These concepts included ideas that evolved throughout the Renaissance, such as harmony, golden ratio, naturalism, anatomical studies, linear perspective, aerial perspective, and chiaroscuro (Janson et al., 1997). tested whether artists in the Early Renaissance had indeed complexity as a possible aesthetic value. To perform this test, Aleem et al. (2017) measured Complexity of Order 1 (Equation 6) and Complexity of Order 2 (Equation 7) in portraits from the period. These authors compared the results with the same complexities from modern spontaneously obtained portraits. Spontaneous and Early-Renaissance portraits with the highest Complexities of Order 1 were equivalent. However, the highest Complexities of Order 2 were larger in Early-Renaissance portraits than in modern spontaneous ones. Hence, Renaissance artists appear to put so much value in amount of information that they made portraits with more complexity than in everyday life.

A possible counterargument to amount of information subserving aesthetic values emerges from the work of Franke (1977). He argued that working memory cannot take in more than 16 bits/s of visual information. He then proposed that artists should provide information along these lines for their works to be aesthetically pleasing. With such low amounts of information, one could think that increasing them further would be ineffectual in raising aesthetic value. However, Franke was discussing, “the brain can consciously process no more than 160 bits at a time.” Therefore, because the literature places aesthetic values mostly in the subconscious realm (Ramachandran and Hirstein, 1999; Brown et al., 2011; Redies, 2015), Franke’s arguments do not derail the belief that amount of information supports aesthetic values.

### When More Information Reduces Aesthetic Values

**FIGURE 4:**
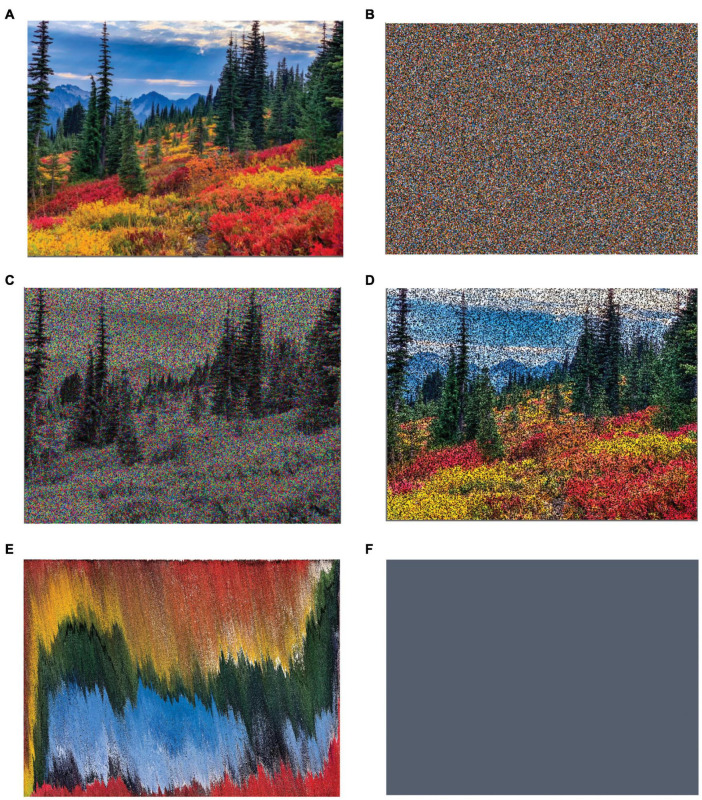
Natural image modified to have too much or too little information. **(A)** The original image is a of a forest during spring. **(B)** Image with the Pixels of the Original Scrambled. **(C)** Image with the Hues of the Original Randomized. **(D)** Image with the Intensities of the Original Randomized. **(E)** Image with the Pixels of the Original Moved Near Other Pixels with Similar Color and Intensity. **(F)** Monochromatic Image with the Mean Color of the Original. Most people like the original image more than versions with too much spatial **(A)**, chromatic **(B)**, or intensity **(C)** complexity (amount of information). Most people like the original image more than versions with too little spatial **(E)** or generic **(B)** complexity. These conclusions are statistical and one cannot generalize them to all people. Some people, specially those educated in modern art, may like the images like **(C)** or **(E)** more than the original. Images used from Shutterstock royalty-free, by Dmitry Kovba: Washington, USA: Fall colors at Paradise area at Mount Rainier National Park (https://www.shutterstock.com/image-photo/washington-usa-fall-colors-paradise-area- 1202207872).

Increasing amount of information through complexity does not always result in an increase of aesthetic preferences. As Figures 4A–D illustrates, too much information does not necessarily make a scene more pleasant. More generally, aesthetic preference drops when complexity becomes too high. Examples of situations when this drop occurs include music (North and Hargreaves, 1995; Gordon and Gridley, 2013; Chmiel and Schubert, 2017), line drawings of house facades (Imamoglu, 2000), and language sequences and random shapes (Munsinger and Kessen, 1964). Miles et al. (2021b) have extended this conclusion from complexity to surprise in musical harmony. They showed that two forms of very high amounts of surprise reduce aesthetic preference: absolute and contrastive surprise. The first is the total amount of unusual information and the second is the contrast in unusual information between two consecutive sections in a musical piece.

This decline of aesthetic preference with increasing complexity might at a first suggest that amount of information does not support aesthetic values. Further evidence apparently making the same suggestion comes from a study of complexity in portrait paintings of the Renaissance (Correa-Herran et al., 2020). That study revealed that complexity declined as the Renaissance progressed. This trend continued in some art movements as the history of art progressed, arguably reaching in Cubism, Constructivism, and Suprematism (Henderson, 2009). These movements made art with very low Shannon-entropy complexity.

#### Inverted-U-Shape Dependence of Aesthetic Preference on Complexity

If amount of information underlies aesthetic values, then why would it decline as the history of art progresses? We address part of the answer to this question in Section “Is Our Definition of Value Compatible With Aesthetic Values?”. In that section we state, “The dependence of value on, say, variables like amount of symmetry or information, may be a complex, non-linear manifold.” Thus, a value function depending on complexity does not always have to rise or be positive as it increases. The value function must predict value correctly, whether it is good or bad. As we now review, a complex value function capturing the good and bad of amount of information seems to be what the brain is using.

Because aesthetic preference drops when complexity becomes too high and preference also rises with complexity (Figure 4, compare panels A, E, and F), the full reported behavior is often of an inverted-U shape. Thus, preference often rises with complexity at low complexities and then falls at high complexities. The mathematical model in Equation 8 is designed to capture this rise-and-fall behavior, which was first predicted by Berlyne (1971). Much of the evidence for the fall of preference with complexity in Section “When More Information Reduces Aesthetic Values” was for this behavior (Munsinger and Kessen, 1964; North and Hargreaves, 1995; Imamoglu, 2000; Gordon and Gridley, 2013; Chmiel and Schubert, 2017; Miles et al., 2021b). Other reports showing this inverted-U-shape behavior include ones for car images (Chassy et al., 2015), color combinations (Tsutsui and Ohmi, 2011), and music (Beauvois, 2007; Delplanque et al., 2019). Importantly, the position of the peak of this behavior depends on the category of objects under study (Sun et al., 2014).

### Amount of Surprise Over Temporally Integrated Expectations

Shannon entropy is the mean amount of surprise in the population of inputs (see Section “Degree of Surprise Given Temporally Integrated Expectations”, and Equations 5 and 7). Consequently, although Shannon entropy and amount of surprise are not the same thing (Figure 2), they have a strong connection. This leads to the questions of whether they share some properties like the inverted-U-shape relationship with preference, and whether they have important differences. The purpose of this section is to answer these questions. Because the study of amount of surprise has focused on music, we will concentrate on it here.

Meyer (2008) was the first to suggest that music elicited a positive aesthetic outcome by employing satisfied or violated expectations. These ideas around expectations and the notion that music always implied “listening ahead” (Margulis, 2007) were expanded by Huron (2008). Meyer’s satisfied expectations leads to the concept of familiarity, which has been central to the studies of emotions elicited by music (Pereira et al., 2011; Madison and Schiölde, 2017). However, as discussed above, the most important idea for this article is that of violated expectations, that is, surprise, which has been studied as a type of amount of information. Many studies have by now shown that surprise indeed leads to musical pleasure (Kellaris and Kent, 1993; Miles et al., 2017, 2021a; Cheung et al., 2019; Shany et al., 2019). Hence, musical amount of surprise has the same effect as Shannon entropy in this regard (see Section “When More Information Increases Aesthetic Values”). This result shows correspondence to the rising portion of the inverted-U-shape behavior for musical surprise, but a systematic demonstration of the falling portion is still lacking. Nevertheless, some initial results suggest that the correspondence exists. Miles (2018) and Miles et al. (2021b) have studied the dependence of aesthetic preference on the amount of harmonic surprise. They found a positive correlation when this amount is in the range of the Amounts of Surprise found in popular music. However, when the amount of surprise exceeds these values, preference stops growing, turning downward. Thus, just like with amount of information, people do not appear to like too much surprise.

However, in two ways, surprise does not yet have immediate correspondence with complexity results: First, both absolute and contrastive surprise appear to affect preference. Absolute surprise is the average amount of surprise in a musical sample, for example, in a chorus or verse section of a song. Listeners probably like this kind of surprise because it suggests new information (Meyer, 1957), thus probably being valuable to them. In turn, the contrastive surprise is the change in amount of surprise in two consecutive sections of the musical sample. In an initially perplexing explanation, a contrastive-surprise boost of aesthetic preference occurs because the brain may consider surprise as bad. This is so because high amount of surprise may indicate to the brain a failure of the prediction ability for the data under consideration. But why would something that is bad increase aesthetic preference? The answer is Huron’s notion of contrastive valence, in which part of a listener’s musical pleasure is due to a discharge from the tension caused by surprise (Huron, 2008). Meyer expressed a similar idea by proposing a “determinate meaning” from the association between antecedents and consequences in music (Meyer, 1957, 2008). Behavioral and socio-statistical studies have provided evidence for the positive effects of absolute and contrastive surprise in musical harmony (Miles et al., 2017, 2021a,b). In neuroscience, support for absolute-surprise effects comes from studies of release of dopamine in musical sections known to evoke “chills” (Salimpoor et al., 2011). As for the contrastive-surprise effect, neuroscience support comes from multiple directions (Patel et al., 1998; Koelsch et al., 2001; Maess et al., 2001; Tillmann et al., 2003). They reveal that the brain computes harmonically unexpected events in music similarly to language syntactic errors.

**FIGURE 5:**
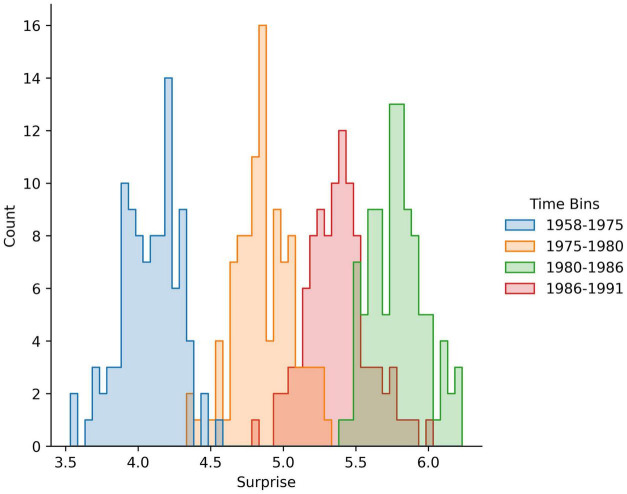
Amount of surprise uses temporally integrated expectations. These schematic histograms are simulated from the data in Figure 1 of Miles et al. (2021a). The simulations assume Gaussian distributions of absolute amount of surprise, with the mean and standard deviation as in Miles et al. (2021a) results. The simulations also assumed a sample of 100 songs per distribution. The average amount of surprise (in bits) is relative to the chord distribution of August 1958 to January 1975. The analyses performed by Miles et al. (2021a) showed increased amount of harmonic surprise over time, specially for songs in the top of the Billboard Top 100.

Second, amount of surprise is measured over temporally integrated expectations (see Section “Degree of Surprise Given Temporally Integrated Expectations”). Thus, in the computation of the amount of harmonic surprise, the brain compares the statistics of the sample with the statistics of a population integrated over many years into the past (Miles et al., 2021a). The logical conclusion of this finding is that something surprising a few years ago is now part of the underlying statistics and thus, not surprising anymore. Thus, what constitutes surprise keeps changing over time (Figure 5). This idea has been termed the Inflationary-Surprise Hypothesis (Miles et al., 2021a). This is a powerful hypothesis that explains why the properties of popular music continuously evolve over time (Mauch et al., 2015). The same inflationary growth may happen for amount of information as an aesthetic value, but no studies of the underlying temporal integration have taken place. On the contrary, studies of amount of information tend to use instantaneous statistics, such as the distribution of properties in paintings (Aleem et al., 2017; Correa-Herran et al., 2020). However, significant hints exist that something like the Inflationary-Surprise hypothesis also applies to amount of information as an aesthetic value. As shown in Section “When More Information Reduces Aesthetic Values,” the amount of complexity has changed over the course of the history of art. These changes are complex, including up-and-down variations, and moments of phase transition (Correa-Herran et al., 2020). An analysis of the changes by Correa-Herran et al. (2020) have suggested that mechanisms akin to the Inflationary-Surprise are at play.

### Why Aesthetic Values Show an Inverted-U-Shape Dependence on Amount of Information

The best published review of the various arguments for why aesthetic values show an inverted-U-shape dependence on complexity is that by Van Geert and Wagemans (2020). In this section, we touch upon their main arguments, discuss them, and expand on the ideas. Much of the Van Geert and Wagemans review is about why results in the literature appear inconsistent on the complexity-aesthetic-preference behavior. Some studies suggest that aesthetic preference rises with complexity (see Section “When More Information Increases Aesthetic Values”). Others show the inverted-U-shape behavior (see Section “When More Information Reduces Aesthetic Values”). One obvious explanation is that in some studies, the range of complexities was not broad enough to capture the falling portion of the inverted-U shape. This explanation is part of a broader one seeing studies as divergent in the kinds, amounts, and ecological legitimacies of stimuli (Marin and Leder, 2013). Furthermore, studies have used a wide variety of definitions, measurements, and manipulations of complexity (Nadal et al., 2010). We agree with these complications, but we will assume for now that an aesthetic value based on amount of information exhibits an inverted-U-shape behavior because it appears in most published studies.

Van Geert and Wagemans also review two theoretical frameworks for aesthetic preferences that are directly relevant for understanding the inverted-U-shape behavior. First, the Processing Fluency Theory, which we discuss in the *Introduction*, would appear to predict a decrease in preference with increasing complexity. The reason for this decrease would be that increased complexity would require increased processing, reducing fluency. However, as we also discuss in the *Introduction*, certain circuitries in the brain have evolved to process as much information as possible. Consequently, the brain is designed to process certain kinds of complex information fluently. For example, the brain processes fluently complex stimuli with inner recurrence, such as fractals (Joye et al., 2016). Second, Van de Cruys and Wagemans (2011) have suggested to use a predictive-coding perspective to understand aesthetic appreciation. According to this perspective, high unpredictability because of high complexity would lead to unpleasantness or confusion, and thus, low aesthetic preference. Hence, this perspective accounts for the declining portion of the inverted-U shape. For the rising portion, Van de Cruys and Wagemans suggested that high predictability would cause boredom. Although this suggestion is fine and consistent with the ideas of Arnheim (2010), it doesn’t address why too much predictability is bad. The Processing Fluency Theory also suffers the same problem with high predictability.

**FIGURE 6:**
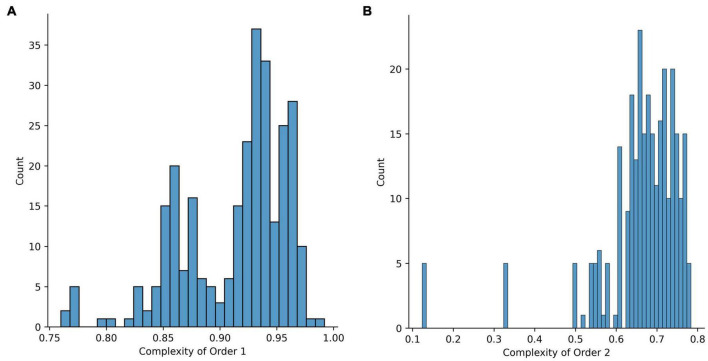
Histograms of complexity of natural images. **(A)** Complexity of Order 1 (Equation 6). **(B)** Complexity of Order 2 (normalized version of Equation 7). We obtained the 286 images for these histograms from the UPenn Natural Image Database, using Albums cd06, cd09, cd12, cd15, and cd23 (Tkačik et al., 2011), which contained no human-made objects. The histograms predominantly exhibit a rise-then-fall inverted-U-shape behavior. Only one notable exception to this behavior is observed in these histograms. Their gross behavior is more interesting than a simple inverted-U-shape, exhibiting a slight bimodality. This bimodality may indicate that images from different environments have dissimilar distributions of complexities. In support of this idea, the very low Complexities of Order 2 in **(B)** are due to some shots of cloudless skies.

A way to address the problem with high predictability is to consider that given limited resources in the brain (see Section “Introduction”), focusing on survival-relevant information as the basis for aesthetic preferences may be important. Relevant information would likely come from nature (Svozil, 2005). So, we must consider the distribution of amount of information in natural sensory stimuli. As the example in Figure 6 illustrates, the distribution of complexities in natural images may have an inverted-U shape. Therefore, when evolution designs brain circuitries to capture as much relevant information as possible, the focus should be on intermediate complexities. Accordingly, boredom with high predictability may just be an indication that the stimulus has an unnaturally low amount of survival-relevant information.

### Fisher Information: Appraisal of Brain Models and Inverted-U-Shape Behavior

Fisher information has not been in the mainstream of the research on aesthetic values and thus, we have little to review. However, we include this section on Fisher Information because it may explain much about aesthetic preferences. In particular, this section makes two points about Fisher Information: (1) the types of information that it conveys to the brain can be so valuable, as to make them good candidates to support aesthetic values. (2) The way that Fisher Information conveys knowledge to the brain may be compatible with the inverted-U-shape behavior without any further assumptions.

**FIGURE 7:**
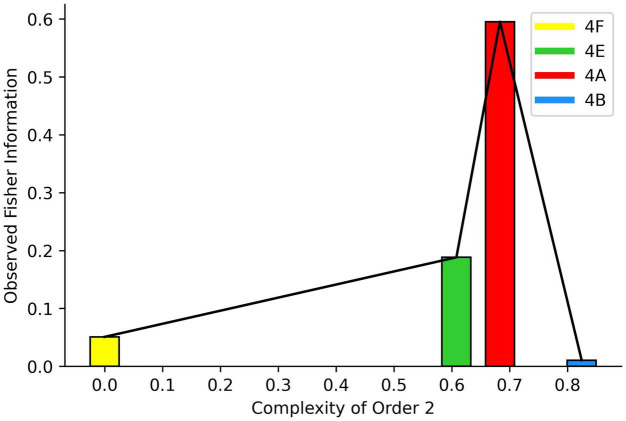
Observed Fisher information as a function of complexity of order 2 for visual images in Figure 4. The complexities of order 2 in the horizontal axis are from four panels of that figure as indicated by the legend. The likelihood function for the Fisher Information in the vertical axis was that of Equation 9, where *P*_*L*_ was Gaussian with standard deviation 1. The value function input to the likelihood function was that of Equation 8. For that equation, we used *w*_1_as the free parameter. In turn, *w*_2_ = 0.69 and *w*_3_ = 0.09 were constants of the equation as determined by fit around the main peak in Figure 6B. Finally, we considered Equation 8 for the fully motivated system, that is, *m*(*t*) = 1. The results based on these equations reveal that the original figure (red) yields the optimal Fisher Information, with it falling when the Complexity of Order 2 is too low or too high.

Fisher Information is a good candidate to support aesthetic values for two reasons (see Section “Amount of Information”): first, it can help the brain determine how much sensory data it must integrate during the learning or adaptation process. Figure 2B shows that when the Fisher Information is high, the brain has fine access to model parameters, thus requiring little integration. The opposite happens when the Fisher information is low. Second, Observed Fisher Information could provide a rapid test of whether an internal cognitive model is good. Such models have been proposed for many brain functions. In the visual domain, examples of such models include, color and motion perception, and geometry, material, and lighting sensing (Kersten et al., 2004; Knill and Pouget, 2004). With other senses, examples include models for multisensory integration, sensory-motor learning, and auditory scene analysis (Knill and Pouget, 2004; Colombo and Seriès, 2012; Cusimano et al., 2018). If incoming sensory signals are too hard to explain by a model that should explain them, it must be wrong. Consequently, if the Observed Fisher Information is too often close to zero (see, for example, Figures 4B,F, 7), the brain has an alarm about the possible inadequacy of the model.

Another reason to include Fisher Information in the conversations about aesthetic values is that it provides a natural way to explain the inverted-U-shape behavior. As Figure 7 shows, the amount of Observed Fisher Information is relatively high for the original image in Figure 4A. In contrast, the Observed Fisher Information is lower for transformations of the image with low (Figures 4E,F) or high (Figure 4B) Complexities of Order 2. The reason for the low Observed Fisher Information with low complexity is that such images have little to say about the parameters of the model. Similarly, when the complexity is too high it cannot inform much about these parameters. Hence, the outcome of the Fisher Information calculation is tightly related to the statistics of natural sensory stimuli, as illustrated by Figure 6. This conclusion is not surprising because Fisher Information captures information pertinent to the brain. Because the brain has limited resources it must focus on information relevant for survival (Ayres, 1997). And such kind of information must be statistically natural (see Section “Introduction”). Finally, the Observed Fisher Information results in Figure 7 also have a relation to the predictive-coding perspective of Van de Cruys and Wagemans. (2011). Fisher Information is not optimal for complexities that are too high because they are unnatural. Therefore, the limited brain does not have the right circuitries to perform computations with too much information.

### Recapitulation of Section Amount of Information as a Possible Aesthetic Value

In this section, we explore the relationship between aesthetic preference and amount information. One of the main findings is that visual aesthetic preference seems to rise and then fall with complexity, giving rise to an inverted U-shaped behavior. When we consider musical surprise and how it relates to preference, studies suggest that an equivalent inverted U-shaped relationship also emerges. However, musical surprise exhibits additional properties due to its temporal nature. For example, people seem to like contrastive surprise, which tells us that we have failed to predict something. Researchers theorize that this may be due to the moment after the surprise, serving to release the listener from built up tension. An additional difference due to the temporal nature of music is that surprise, unlike visual complexity, is measured through temporal integration. Finally, this section returns to the inverted U-shaped behavior of complexity and surprise, and consider several explanations for it. In particular, we consider both cognitive-processing theories and predictive-processing accounts of this behavior. The latter suggests that too little complexity or information may be disliked because it provides little to predict. Likewise, too much complexity may be incomprehensible, also leading to little useful information. In this context, Fisher Information may explain the inverted U-shape behavior without further assumptions. Given our evolutionary history, the cognitive models that our brain uses for perception are based on statistical information about the natural world. These properties appear to exhibit a peak around an optimal complexity, causing Fisher Information also to peak at intermediate values.

## Discussion

### Amount of Information Supports Aesthetic Values

In this article, we have asked whether amount of information supports aesthetic values. We believe that the preponderance of the evidence presented allows us to answer this question in the positive. However, as the article also emphasizes, this is not an easy question to answer. It turns out that to provide such an answer, one must first address the issue of whether aesthetic values are an objective reality. As pointed out in Section “Is Our Definition of Value Compatible With Aesthetic Values?”, for some scholars, aesthetic values are socially constructed and thus, they should be discredited as true entities. The arguments that we use in that section to rebut the purely social-construct idea involves genetic predisposition and the presence of aesthetic preferences early in development. We thus conclude the arguments by repeating an statement of Krentz and Earl (2013). They say, “[these] results demonstrate that appreciation for … [certain] features of artworks … are biologically driven.”

If we accept that aesthetic values are real, we must ask whether amount of information underlies at least one of them. Again, this is not an easy question to answer because the historically prevalent idea is that aesthetic values are about beauty and thus have positive valence. However, aesthetic preferences exhibit an inverted U-shape dependence on complexity, a proxy to amount of information. Consequently, to accept amount of information as the basis of aesthetic values, we must abandon the idea that they only reflect positive valence. Fortunately, as we again point out in Section “Is Our Definition of Value Compatible With Aesthetic Values?”, this idea is increasingly in disfavor. Its replacement is the broader naturalistic or everyday aesthetics. Thus, the Kantian notion of disinterested aesthetics may perhaps be subsumed by a neuroscientific view of aesthetic values (Brown et al., 2011; Aleem et al., 2019).

Given the complex dependence of preference on amount of information, what are the best pieces of evidence for the latter supporting aesthetic values? We ask this question because maybe amount of information is the wrong variable to study, giving rise to an irrelevant behavior. This article raises two pieces of evidence here for some aesthetic values having the support of amount of information: First, the inverted-U-shape behavior follows the statistics of the natural world (Figure 4) even when they are messy (Figure 6). This is compatible with the limited-capacity brain evolving circuits matched to natural, survival-dependent information (see Section “Fisher Information: Appraisal of Brain Models and Inverted-U-Shape Behavior”). Second, in the Early Renaissance, artists emphasized amount of information (Figure 3), making portraits with more complexity than natural (see Section “When More Information Increases Aesthetic Values”). Thus, amount of information was so compelling that pushed artists beyond reality even they aimed for naturalism.

However, this last piece of evidence is not meant to say that art is just about aesthetics or driven mostly by amount of information. Far from it, art straddles far beyond aesthetics (Carroll, 2001; Welsch, 2003; Pinney and Thomas, 2020). The same goes for the relationship between amount of information and art. As pointed out in Section “When More Information Reduces Aesthetic Values,” visual artists have occasionally abandoned amount of information in favor of other tools. Similarly in music, complexity has occasionally been elevated beyond the point of comprehensibility (Jonathan, 1988; Lerdahl, 1988) both to imply transcendence from art and to overwhelm the listener (Jonathan, 1988; Lerdahl, 1988; London, 2009).

### Why Do Aesthetic Values Use Amount of Information?

The brain gains multiple advantages when using amount of information as the basis of aesthetic values. The most obvious advantage is that the amount of information can signal to the brain how much it can hope to find in the inputs. Another advantage discussed in Section “Information Versus Amount of Information” is that with more information, the brain learns that it must devote more resources to process the incoming sensory signals. Thus, amount of information may be valuable as a type of attentional trigger. Fisher Information can also signal on needed resources. In Section “Amount of Information,” we proposed that a possible function of Fisher Information is helping the brain decide on the resources necessary to establish good parameters for its cognitive models. We also proposed an advantage of Observed Fisher Information. It could help the brain determine whether its current (parametric) believes are appropriate for the present situation (Figure 7).

Other advantages can follow by using samples with surprising information (Figure 1) because high amount of surprise indicates new use of information and thus, something that the brain can learn. However, as emphasized by Barto et al. (2013), we should not confuse surprise and novelty. Surprise is unexpected use of known information, thus causing an emotion that is different from what we get when exposed to something new. Interestingly, surprise also appears to exhibit an inverted-U-shape behavior (see Section “Amount of Surprise Over Temporally Integrated Expectations”). Because of this behavior, some artists have been attacked in high moments of creativity. A famous example was Igor Stravinsky’s The Right of Spring. Many consider this composition “undoubtedly the most famous composition of the early 20th century” (Grout and Palisca, 1981). However, the level of surprise of this composition was so high that at the premiere, mocking laughter met the Introduction and a pandemonium happened during the performance (Stravinsky, 1962).

One of the best analysis of why information is so advantageous is due to Israel and Perry (2012). They write, “What underlies the phenomenon of information is the fact that reality is lawlike; that what is going in one part of reality is related to what is going on in some other part of reality, by laws, nomic regularities, or as we shall say, constraints.” Hence, information is a consequence of the laws of nature. One should thus not be surprised that information is now firmly established in the laws of the natural sciences themselves (Landauer, 1991, 1996; Frieden, 2000, 2004; Barbieri, 2016). Consequently, because amount of information supports aesthetic values, art exhibits lawlike regularities (Graham and Redies, 2010).

The lawlike nature of information relates to another important proposal for what aesthetic appreciation is. Schmidhuber (2009) proposes that because the brain has computational limitations, an important task for individuals is to learn to compress incoming sensory data. Thus, the processed data become in a way “simpler and more beautiful.” As Schmidhuber puts it so eloquently, “[learning to compress] motivates exploring infants, pure mathematicians, composers, artists, dancers, comedians, yourself, and (since 1990) artificial systems.” Using the line of argumentation in this article, we propose that data comprensability is another advantage that amount of information endows the brain. It gets lawlike knowledge from information.

### Where in the Brain

Much neuroscience research has been performed in recent years on aesthetic values and amount of information. An especially relevant meta-analysis of neuroimaging examined common characteristics of aesthetic evaluation across different senses (Brown et al., 2011). The analysis found universal mechanisms for aesthetic appraisal with the four most concordant brain regions of activation being the orbitofrontal cortex, anterior insula, anterior cingulate, and the ventral basal ganglia. Roughly speaking, the orbitofrontal cortex is a sensory integration area of the brain capable of value-based decision making (Kringelbach, 2005; Fellows, 2011; Wallis, 2012). In turn, the anterior insula receives inputs from internal parts of the body, being specially important in bringing motivation to actions (Craig, 2003; Zaki et al., 2012; Wager and Barrett, 2017). Next, the anterior cingulate is an area responsible for processing behavioral errors (Carter et al., 1998; Bush et al., 2000; Botvinick et al., 2004). Finally, ventral basal ganglia have many roles, with the most important for us being the mediation of reward-based learning (Schultz et al., 2000; Packard and Knowlton, 2002; Yin et al., 2008). The central importance of reward appears in other imaging studies of aesthetics and appraisal (Lacey et al., 2011; Vartanian and Skov, 2014; Wang et al., 2015).

Some of the same brain areas may be involved in how amount of information contributes to aesthetic appreciation. In Section “Amount of Surprise Over Temporally Integrated Expectations,” we discussed how the release of dopamine in the basal ganglia helps to evoke “chills” when the amount of musical absolute surprise was high. We also discussed that the computation of the musical contrastive-surprise effect is like that of language syntactic errors. More evidence has accumulated in this direction in neuroscience-of-music research. Research by Shany et al. (2019) revealed associations of music-induced pleasantness with surprise in the activity of the nucleus accumbens, a component of the ventral striatum of the basal ganglia. In turn, Cheung et al. (2019) found that activity in the amygdala, hippocampus, and auditory cortex reflected chords eliciting pleasure when they deviated from what the listener had expected. These areas also elicited pleasure when the chords conformed to expectations. The participation of these brain areas is not surprising because music can modulate activity in structures involved in emotion. These areas include the amygdala, nucleus accumbens, hypothalamus, hippocampus, insula, cingulate cortex, and orbitofrontal cortex (Koelsch, 2014). For Cheung et al. (2019), the nucleus accumbens only reflected the uncertainty of those emotions. This is important because the nucleus accumbens was previously associated with the processing of pleasure from music (Salimpoor et al., 2013). In addition, the reflection of uncertainty in the nucleus accumbens is important for reward learning processes.

Because amount of surprise can only work over a baseline of stored information, one also must know where in the brain this storage is and how it works. Two distinct neural mechanisms are implicated in the learning of musical information and thus, familiarity (Miranda and Ullman, 2007). One involves the processing of statistically learned information about musical harmony. The other involves the processing of explicitly learned information that reflects familiarity with a specific piece of music (see Section “Amount of Surprise Over Temporally Integrated Expectations”). The superior temporal cortex is believed to accumulate templates of sound events that a person learns over time (Peretz et al., 2009; Salimpoor et al., 2015). In evidence, electrical stimulation of the superior temporal cortex provokes musical hallucinations (Penfield and Perot, 1963). Moreover, augmented activity in this region has associations with imagery (Herholz et al., 2012) and familiarity of music (Peretz et al., 2009; Groussard et al., 2010). These results suggest that this region stores previously heard auditory information. Therefore, auditory information attained in this region may provide the basis for anticipation during music listening. This is consistent with the finding that enjoying new music has associations with augmented activity in the nucleus accumbens and its strong connectivity with large clusters of the superior temporal cortex (Salimpoor et al., 2013).

Besides the orbitofrontal and the superior temporal cortices, another cortical area appears to participate in valuation in the brain. Bartra et al. (2013) performed a meta-analysis of functional brain imaging in which people assessed stimulus value. They found that only the ventromedial prefrontal cortex and ventral striatum appeared to track value. Other areas tracked related but non-specific features, such as salience or arousal response.

Interestingly, some neurodegenerative disorders impair the ability of the brain to process musical surprise. A recent study explored Alzheimer’s disease and some forms of dementia in regard to this impairment (Benhamou et al., 2021). Alzheimer’s disease associated with normal deviant detection accuracy. However, behavioral and semantic variant frontotemporal dementia syndromes are associated with compromised syntactic and semantic deviant detection accuracy. These dementia impairments are further corroboration that the brain appears to have dedicated mechanisms for the detection of surprise and thus, large amount of unusual information.

### Shannon Entropy Versus Fisher Information

In this article, we have suggested that amount of information in the form of either Shannon entropy or Fisher Information can support aesthetic values. But would one of them be better than the other or could them both serve different aesthetic values? As seen in Sections “Amount of Information” and “Degree of Surprise Given Temporally Integrated Expectations,” Shannon entropy has been the historically favorite method to quantify amount of information. This favoritism is due in part to the success of Information Theory. Furthermore, Shannon entropy can measure all available information regardless of how the brain uses it. However, we can see at least two of disadvantages to Shannon entropy: first, it does not on its own capture the inverted-U-shape behavior (Figure 4). Instead, if the brain uses Shannon entropy for its aesthetic values, a special value function (Equation 8) must be in place after the computation of complexity to capture the behavior observed in nature (Figure 6). Second, even if the brain wanted to have access to all the information in the external world, that would not be possible. Our intention is not to say that the brain wants all information in the world. We just want to emphasize that Shannon entropy does not quantify the information useful to the brain. As we discussed in Section “Fisher Information: Appraisal of Brain Models and Inverted-U-Shape Behavior,” because the brain has limited resources, it focuses on information relevant for survival. The circuits that the brain has evolved have designs optimized to certain kinds of information.

In turn, Fisher Information does not suffer from these disadvantages. To begin with, it naturally captures the inverted-U-shape dependence of aesthetic preference on amount of information (Figure 7). This is possible because Fisher Information emerges from models of the brain, which itself has evolved to be in tune with survival-relevant information (Ayres, 1997). Thus, the brain could possibly use a single stage of computation instead of two by incorporating the value function (Equation 8) in the likelihood function (Equation 9). And by employing Fisher Information, the brain would not even be aiming to use all the information in the world. However, Fisher Information has at least two disadvantages. First, it has not been explored systematically in the aesthetic-value literature, so we have no clear idea of what the limitations are. Nevertheless, Fisher Information is gaining much traction in science in general (Frieden, 2000, 2004) and neuroscience in particular (Brunel and Nadal, 1998), raising hopes that better tools and deeper understanding of the potentials and limitations of Fisher Information are coming. Second, Fisher Information, but not Shannon entropy, is model dependent. To employ the former in brain research, one must have a clear idea of what the system is trying to achieve with the information. Thus, in this regard, Fisher Information requires more assumptions than Shannon entropy, being thus less elegant.

To overcome the first disadvantage of Fisher Information highlighted in the last paragraph, we propose the following five-step research program in the visual/auditory domains: (1) Obtain spontaneous (not posed) images/sounds from different environments relevant to humans (natural and urban). (2) For each domain, measure the distributions of complexities according to specific variables (for example, pixel intensities, chromatic contrast, or inter-beat interval). (3) Build simple parametric models to capture these distributions, with as few parameters as possible. We provide an example of this step in the transition from Figures 6, 7. We model the example in Figure 6 with a Gaussian distribution, fitting only the main peak for simplicity. The random variable in this model is Complexity of Order 1. The free parameter is the mean of Gaussian (as we keep the standard deviation constant, also for simplicity). Because each image in Figure 4 has its own Complexity of Order 1, we can then compute the Observed Fisher Information for each image using the Gaussian model. (4) Develop a set of artificial images with which to conduct cognitive-preference experiments. (5) Measure Fisher Information measures from each of these images to compare with the experimentally obtained preferences. Our laboratory is currently undertaking such a five-step program to test the applicability of Fisher Information for aesthetic values.

### Individuality

Individual differences play an important role in determining aesthetic appreciation (Jacobsen, 2004; Vessel and Rubin, 2010; Güçlütürk et al., 2016; Van Geert and Wagemans, 2020). The first to study systematically the mechanisms of such differences was Eysenck (1942), who identified two general and two unique factors of individuality. The two general factors related to taste, and to order and complexity. Eysenck related the order and complexity factors to introversion and extroversion, with the latter leading to preference of stimuli that were more complex, that is, with higher amount of information. Regarding the two “unique” factors, that is, peculiar to each observer, he distinguished “specific” and “error” factors. Specific factors include those based on private associations and experiences, while error factors indicate those that show intraindividual variability. Recent theoretical studies on the learning of aesthetic values explore these factors (Aleem et al., 2020; Grzywacz, 2021).

Other factors beyond those identified by Eysenck play a role in individuality. One such factor is individual differences in abilities, such as having good or bad vision, hearing, and memory (Munsinger and Kessen, 1964; Chevrier and Delorme, 1980; Sherman et al., 2015; Van Geert and Wagemans, 2020). For example, if one cannot see details because of poor vision, the amount of information becomes less relevant to aesthetic preferences. Individual aesthetic values are also affected by differences in personality (Van Geert and Wagemans, 2020). For instance, Openness to Experience is a key personality trait related to preference for art in general (Chamorro-Premuzic et al., 2010). Not only that, but Openness to Experience is the personality trait most consistently found to relate to aesthetic preferences for complexity in music (Rentfrow and Gosling, 2003) and visual art (Chamorro-Premuzic et al., 2010). The theoretical works mentioned above also emphasize this openness-to-experience personality axis in the development of complexity preference (Aleem et al., 2020; Grzywacz, 2021). However, in their studies of preference learning, these authors use the terminology risk-aversion and risk-taking. Besides personalities traits, individuality in aesthetic preference for amount of information is influenced by education level (Frances, 1976), expertise (Munsinger and Kessen, 1964; Orr and Ohlsson, 2005; Nadal Roberts, 2007), and creativity (Taylor and Eisenman, 1964). Finally, demographics and culture play roles in individuality. For example, sex is a significant determinant of complexity preference, with females tending to prefer more complexity than males (Eisenman, 1967). In terms of culture, we point out the work of Curtis and Bharucha (2009) who found that the musical culture that an individual grows up with has an influence on the understanding and perception of music later in life. This effect is so strong that an individual’s perception of, and enjoyment of, a new musical piece is dependent on music already heard (McDermott et al., 2016).

A final factor worth mentioning in the understanding of individuality is competition between aesthetic values. Aleem et al. (2017) pointed out that while certain variables supporting aesthetic values increase others must decrease. For example, a painting with high symmetry or balance tend to have low Complexity of Order 2 because these properties inform what certain parts of the canvas look like when one knows about other parts. Thus, artists must choose how to equilibrate their paintings in terms of such co-dependent variables. If an artist chooses to use more symmetry, they know that they would lose complexity. Thus, equilibrating these variables is an individual choice. Not surprisingly, when Aleem et al. (2017) analyzed portraits of the Early Renaissance, they found that each artist positioned their paintings as islands in different positions of the balance-complexity space. Different artists produced different, often non-overlapping islands in terms of balance and complexity. Recent perceptual experiments have confirmed this competition tendency between complexity and symmetry (Pombo et al., 2021), with the latter being a stronger predictor of beauty judgments than the former (Tinio and Leder, 2009). Based on this apparent choice space with variables like symmetry, balance, and complexity, Aleem et al. (2017) generalized and proposed the existence of a neuroaesthetic space in the brain. This is undoubtedly a very high dimensional space. Just by considering complexity, for example, we already have three dimensions in Figure 4, namely, of order 1 and 2, and chromatic. But the complexity of complexity does not stop there. Many authors have noted that visual complexity is a multidimensional concept (Berlyne et al., 1968; Rump, 1968; Kreitler et al., 1974; Chipman, 1977; Nadal et al., 2010), thus creating the certainty that the neuroaesthetic space is indeed very high dimensional.

### Decision Making

People’s thirst for information appears to be insatiable. A demonstration of this thirst is the exponential growth of information technology over the years (Huberman and Adamic, 1999; Denning and Lewis, 2016). The reason for the thirst is complex, but one of the most important factors is that decision-making in all corners of our lives requires information (March, 1987; Blanchard et al., 1988; De Freitas, 2003). In most rationale decision-making, information is used together with our believes to choose a good path to follow (Slovic and Lichtenstein, 1971). Because information is so important, having large amounts of it is beneficial. And, as we conclude in this article, the brain values knowing how much information is incoming. We also argue that the brain uses knowledge on amount of information in many of its decisions. For example, it helps the brain decide both how much neural resources to allocate to process the incoming information and how well the internal cognitive models are doing. In addition, amount of information as a support of aesthetic values helps the brain in practical decision-making in everyday tasks. An example covered in this article is the demonstration by Miles et al. (2017, 2021a) that higher amount of harmonic surprise, that is, higher amount of unusual harmonic information, increases the chance that a listener will decide to buy a song. Another example is the negative correlation between the complexity of a webpage and how beautiful it looks (Michailidou et al., 2008; Tuch et al., 2012; Reinecke et al., 2013). Hence, the amount of information in a webpage influences the decisions on what it is selling. And we even covered the influence of amount of information in the interface design for smartwatches (Wang and Hsu, 2020). More information causes emotional arousal and valence in the shopper, making them decide to buy the watch.

Unfortunately, strong values can also lead to serious mistakes in decision-making and information-based values are not exception. Values, information-based aesthetic ones included, can lead to addiction and thus, impair the decision-making abilities of individuals (Bechara and Damasio, 2002; Bechara et al., 2002; Bechara, 2003). As we suggest in this article, the craving for large amounts of information is an inescapable need that makes the brain perform better in multiple essential decision-making tasks. However, the brain has limitations and thus, has mechanisms to find vital information (Todd, 2007). These mechanisms are important given that and too much information is not only not useful, but could be detrimental. Perhaps this realization will help people find a balance in how much amount of information is enough.

### Concluding Remarks

Our analysis of the literature suggests that amount of information supports an aesthetic value. This support is not simple, with aesthetic preference exhibiting a complex dependence on the amount of information. We suggest that this complexity arises because the statistics of the stimuli arriving from natural world are themselves complicated. Thus, because the brain has finite capacity, it must adjust closely to the survival-relevant features of the incoming information. Hence, aesthetic signals of amount of information may benefit the brain by alerting it to how much it can hope to find in the sensory inputs. The brain can thus prepare itself to devote the right amount of resources to process the incoming sensory signals. The necessity of the brain to prepare itself for the incoming information has two important consequences. First, the brain must have good cognitive models of the world, that is, those focusing on survival-relevant information. Those models must have arisen through evolution, and continue to arise by development and learning. Because survival-relevant aesthetic values would depend on these models, we propose to quantify the relationship between amount of information and aesthetic values through Fisher Information. The most popular alternative, namely, Shannon entropy, captures all the information, even some that are irrelevant. Thus, using Shannon entropy for aesthetic values is probably leading to unnecessarily complex descriptions of behavior. Second, the necessity to learn the right cognitive models and their parameters lead to the prediction of high individuality in terms of aesthetic values. This individuality arises because of the contribution of interoceptive signals, which are different among people. In addition, different socio-cultural contexts are distinct and impart disparate values, thus leading to further individuality through learning.

## Author Contributions

NG wrote the first version of the manuscript and prepared Figure 6. HA collaborated in the latter versions of the manuscript, preparing Figures 1–5, 7. Both authors contributed to the article and approved the submitted version.

## Conflict of Interest

The authors declare that the research was conducted in the absence of any commercial or financial relationships that could be construed as a potential conflict of interest.

## Publisher’s Note

All claims expressed in this article are solely those of the authors and do not necessarily represent those of their affiliated organizations, or those of the publisher, the editors and the reviewers. Any product that may be evaluated in this article, or claim that may be made by its manufacturer, is not guaranteed or endorsed by the publisher.
